# Pulsatile sequential drug release system for cascade tumor deep penetration and differentiation therapy to enhance chemoimmunotherapy

**DOI:** 10.1126/sciadv.adr8001

**Published:** 2025-09-03

**Authors:** Fengxiang Liu, Shipeng Ning, Xia Wang, Xiaoyuan Fan, Bin Wan, Fei Sun, Lili Du, Kefan Shi, Xinpeng Zou, Ruihong Zhu, Mingxing Li, Wenwen Shen, Zhonggui He, Kaiyuan Wang, Jin Sun

**Affiliations:** ^1^ Department of Pharmaceutics, Wuya College of Innovation, Shenyang Pharmaceutical University, Shenyang 110016, China.; ^2^ Department of Breast Surgery, The Second Affiliated Hospital of Guangxi Medical University, Nanning 530000, China.; ^3^ Guangdong Provincial Key Laboratory of Urology, Guangdong Engineering Research Center of Urinary Minimally invasive surgery Robot and Intelligent Equipment, Guangzhou Institute of Urology, The First Affiliated Hospital of Guangzhou Medical University, Guangzhou Medical University, Guangzhou 510120, China.; ^4^ Department of Pharmaceutics, School of Pharmacy, Shenyang Pharmaceutical University, Shenyang 110016, China.; ^5^ Department of Pharmacy, The First Affiliated Hospital of Jinzhou Medical University, Jinzhou 121000, China.; ^6^ Joint International Research Laboratory of Intelligent Drug Delivery Systems, Ministry of Education, Shenyang 110016, China.; ^7^ Departments of Diagnostic Radiology, Surgery, Chemical and Biomolecular Engineering, and Biomedical Engineering, Yong Loo Lin School of Medicine and College of Design and Engineering, National University of Singapore, Singapore 119074, Singapore.

## Abstract

Cancer stem cells (CSCs) and myeloid-derived suppressor cells (MDSCs) contribute to chemoresistance and immunosuppression, constraining chemoimmunotherapy outcomes. Differentiation therapy, aiming to mature CSCs and MDSCs, shows great promise. However, its efficacy is hindered by limited accessibility in hypoxic deep tumor regions. Inspired by the apoptotic body (ApoBD)–mediated deep tumor penetration, we design a pulsatile sequential drug release system with a core-shell structure. The reversible acid-responsive shell protonates and swells in lysosomes to release doxorubicin, inducing lysosomal escape and cell apoptosis. In ApoBDs, it deprotonates and contracts to prevent excessive drug release. After deep penetration via ApoBDs, the hypoxia-responsive core releases all-trans retinoic acid to reverse CSCs and MDSCs, overcoming chemoresistance and modulating the immuno-microenvironment. This strategy targets the heterogeneous distribution of CSCs and MDSCs in solid tumors, enhancing chemo-intervention and immune checkpoint blockade therapy while presenting encouraging potential for cascade deep tumor penetration and differentiation therapy.

## Teaser

Engineered drug delivery systems enable precise spatiotemporal release of anticancer agents for enhanced cancer therapy.

## INTRODUCTION

Till now, chemotherapy has remained the standard of care strategy in clinical settings for tumor treatment, but the introduction of cancer immunotherapy has altered the practice of oncology (*1*–*3*). Among them, immune checkpoint inhibitors (ICIs), especially anti-programmed death-1 antibody (aPD-1), have demonstrated unprecedented success in improving the prognosis for certain cancers (*4*, *5*). Chemoimmunotherapy, the combination treatment that leverages the benefits of chemotherapy and immunotherapy, has become a cornerstone in the multimodal treatment of various cancers. Specifically, chemotherapeutic agents kill tumor cells rapidly and reduce the tumor burden, while immunotherapy triggers systemic and durable antitumor immune surveillance that prevents metastasis and recurrence (*6*, *7*). On the basis of these benefits, several related clinical trials have been launched (*8*). Unfortunately, despite the US Food and Drug Administration’s approval of chemoimmunotherapy combining Nab-paclitaxel and atezolizumab for metastatic triple-negative breast cancer, subsequent clinical trials failed to confirm survival benefits of this combination, with no statistically significant improvement in overall survival (*9*, *10*). Amounting research also underscores that inherent and acquired chemoresistance as well as immunosuppressive environment gravely impair the outcomes of chemoimmunotherapy (*11*, *12*).

The tumor microenvironment (TME) is highly heterogeneous and characterized by hypoxia, forming multiple obstacles to treatment resistance (*13*). Wherein, cancer stem cells (CSCs), a malignant subset of tumor cells with a robust tumorigenic capacity, are deemed crucial in tumor heterogeneity (*14*). Owing to their particularity of entering the quiescent state and up-regulated multidrug-resistant proteins, CSCs develop resistance to chemotherapy, aggravating the risks of relapse (*15*, *16*). Besides, a population of immature myeloid cells known as myeloid-derived suppressor cells (MDSCs) is closely associated with tumor immune escape (*17*). These cells diminish the functions of T cells and prevent their entry into TME by secreting immunosuppressive cytokines [e.g., transforming growth factor–β (TGF-β) and interleukin-10 (IL-10)] and depriving essential nutrients through mechanisms involving arginase-1. Notably, the symbiotic cross-talk between CSCs and MDSCs has been revealed in multiple pieces of evidence (*18*). CSCs facilitate the expansion of MDSCs by producing immunosuppressive factors and exosomes. Concurrently, MDSC-induced activation of signal transducer and activator of transcription 3 (STAT3) and Notch pathways further promotes stemness (*19*, *20*). It has been demonstrated that the sustenance of these inhibitory cell populations primarily relies on hypoxia, as they preferentially accumulate in response to the elevated levels of hypoxia-inducible factor (HIF) (*21*–*23*). Therefore, targeting CSCs and MDSCs is crucial for developing strategies that address the challenges of chemoresistance and immune escape in chemoimmunotherapy.

Differentiation therapy uses agents like all-trans retinoic acid (ATRA) to regulate differentiated gene expression, aiming to eliminate stem-related phenotypes and promote maturation (*24*). It represents a promising therapeutic strategy on account of the tremendous success of ATRA in acute promyelocytic leukemia (*25*). Simultaneously, ATRA has been reported to be effective in reactivating the differentiation program of MDSCs into mature myeloid cells, prompting the investigation of the combined treatment efficiency with aPD-1 in phase 2 clinical trials (*26*–*28*). However, the dense extracellular matrix together with high interstitial pressure collectively contributes to the poor accessibility of differentiation-inducing agents to the CSCs and MDSCs located in the hypoxic regions away from the vasculature, which leads to unsatisfactory outcomes of differentiation therapy in solid tumors (*29*–*31*).

Our previous research found that nanoparticles with strong neighboring effects exhibited enhanced tumor penetration (*32*). Apoptotic bodies (ApoBDs) can serve as reservoirs for residual drugs, amplifying the deep-penetrating advantage of the neighboring effect. These apoptosis-induced ApoBDs can harbor cytoplasm containing organelles and molecular cargos, including remaining drugs, which are further delivered to surrounding tumor cells (*33*, *34*). Generally, intracellular drugs comprise inactivated drugs tightly bound to target sites, active unbound drugs, and inactive prodrugs shielded by the protective groups. The unbounded drugs continue to exert cytotoxic effects during the subsequent permeation process. However, uncontrollable drug release rapidly decreases the proportion of unbound active drugs, thereby impairing ApoBD-based drug delivery. This highlights the need for rational design to achieve on-demand drug release. Motivated by the typical intracellular fate of nanomedicines, which involves translocation from the lysosome to the cytoplasm accompanied by a pH variation from 5.0 to 7.4, we hypothesized that the reversible acid-responsive nanoplatform could prevent the burst drug release (*35*).

**Fig. 1.**
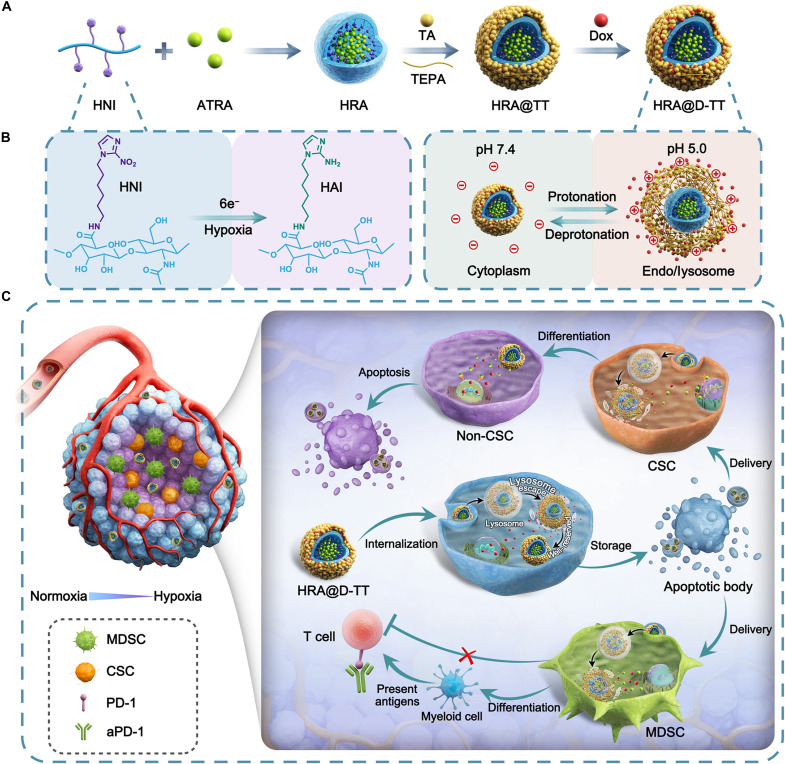
Schematic illustration of HRA@D-TT as a pulsatile sequential release platform for cascade tumor penetration and differentiation therapy. (**A**) The preparation of the pulsatile sequential drug release platform. (**B**) Mechanism diagram of hypoxia-responsive drug release in the inner core and reversible acid-responsive drug release in the outer shell. (**C**) Schematic representation of the pulsatile sequential drug release platform to achieve tumor penetration and subsequently eliminate CSCs and MDSCs for enhanced chemoimmunotherapy. Upon internalization by tumor cells, the low pH within lysosomes triggers protonation and swelling of the shell, facilitating DOX release and lysosome escape. In the cytoplasm, neutral pH conditions cause the shell to shrink, halting drug release and storing the remaining DOX in DOX-induced ApoBDs. After penetrating deeper tumor regions, hypoxia induces the conversion of nitroimidazole groups to aminoimidazole, leading to the release of ATRA. This release promotes the differentiation of CSCs and MDSCs, overcoming chemoresistance and immunosuppression.

Here, we proposed a pulsatile sequential drug release system as a proof of concept for cascade drug penetration and differentiation therapy to overcome therapy resistance (Fig. 1 and fig. S1). The core-shell nanoplatform (denoted as HRA@D-TT), composed of a tannic acid (TA) and tetraethylenepentamine (TEPA) polymerized shell loaded with doxorubicin (DOX), exhibited reversible pH-responsive behavior. Additionally, it featured a nanocore derived from hyaluronic acid (HA) that released encapsulated ATRA responsively under hypoxic conditions. As shown in Fig. 1, upon internalization by tumor cells, the polymer shell underwent protonation and swelling within lysosomes, facilitating the subsequent DOX release and mediating lysosomal escape. In the cytoplasm, it shrunk in response to pH variation, thereby halting drug release. This pulsatile release mechanism enabled the storage of nanoplatforms with residual drugs in DOX-induced ApoBDs, which were disseminated in deep tumor regions through sustained infection of neighboring cells. Facilitated by ApoBD-mediated penetration and the protective nanocore, ATRA was protected from premature leakage in the outer tumor regions and released specifically at the inner hypoxic sites. This targeted release promoted the differentiation of CSCs and MDSCs, thereby reshaping the hostile TME characterized by chemoresistance and immunosuppression. The application of pulsatile sequential drug release addressed the heterogeneous distribution of CSCs and MDSCs, substantially enhancing chemotherapy and boosting the antitumor immune activity of aPD-1 in various tumor models.

## RESULTS

### Confirmation of hypoxia as a driver for CSCs and MDSCs infiltration

HIF-1α functions as an oxygen-dependent transcriptional activator whose regulation is intricately linked to cellular oxygen availability (*36*). Under normoxic conditions, HIF-1α undergoes rapid degradation via the ubiquitin-proteasome pathway, resulting in transient expression. Conversely, hypoxic environments stabilize the HIF-1α subunit, enabling interaction with transcriptional coactivators to modulate its functional activity. Consequently, HIF-1 serves as a primary regulator of numerous hypoxia-responsive genes in oxygen-deficient microenvironments and is frequently used as a canonical hypoxia biomarker (*37*). Within solid tumors, vascular irregularities coupled with accelerated tumor proliferation generate localized oxygen deprivation, leading to pronounced HIF-1α expression in affected regions (*38*). Substantial evidence demonstrated that these hypoxic tumor domains facilitated the expansion of CSCs and MDSCs (*21*, *22*). To confirm the spatial distribution of these immunosuppressive populations, immunofluorescence analysis was performed on 4T1 tumor sections. The selected molecular markers included HIF-1α (hypoxia), CD133 (CSCs), and Gr-1 (MDSCs). As illustrated in fig. S2, HIF-1α signal intensity exhibited a positive correlation with both Gr-1 and CD133 expression, demonstrating significantly enhanced CSC and MDSC infiltration within hypoxic tumor compartments compared to normoxic regions. These findings substantiated that HIF-1α activation augmented tumor cell stemness while simultaneously promoting MDSC recruitment.

### Fabrication and characterization of HRA@D-TT

To construct the nanocore, nitroimidazole-modified HA (HNI) was first synthesized as outlined in a previous study (fig. S3) (*39*). Following purification, the chemical structures (compounds a, b, and c) were subjected to ^1^H NMR analysis (figs. S4 and S5) to confirm the successful synthesis of HNI. The ATRA-loaded nanocore (HRA) was then prepared via the emulsion solvent evaporation method. Subsequently, leveraging the high adhesion and reducing properties of its abundant phenolic hydroxyl groups, TA facilitated the formation of a polymer shell on HRA through a Michael addition–Schiff base reaction with the amino groups of TEPA in an alkaline environment (fig. S6) (*40*, *41*). The successful synthesis of the binary system (TA-TEPA) was evidenced by the additional signals at 2960 and 2852 cm^−1^ in the infrared absorption spectrum, which was ascribed to the C-H stretching vibration from TEPA (fig. S7). Last, the shell-covered nanocore was incubated in DOX solution for 2 hours to produce HRA@D-TT. The molar ratio of DOX:ATRA was optimized to 1:3, as previously determined to provide the most significant therapeutic benefits (*42*). The loading capacities of DOX and ATRA in HRA@D-TT were ~2.57 and 7.62%, respectively.

**Fig. 2.**
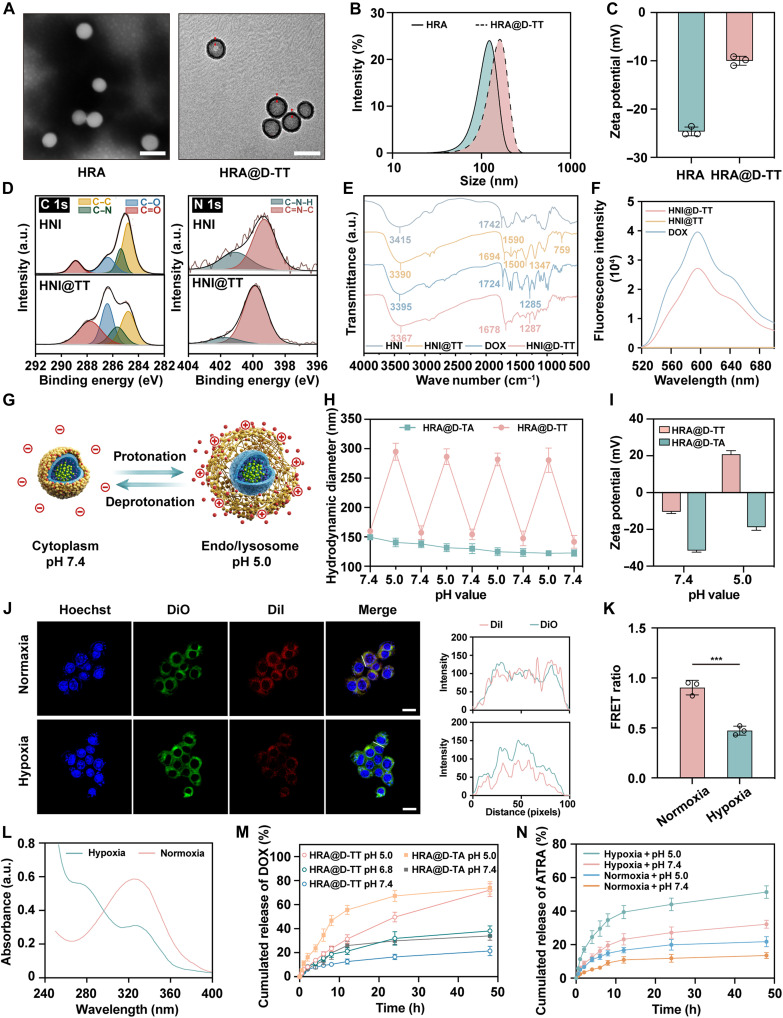
Characterizations of HRA@D-TT. (**A**) TEM images of HRA and HRA@D-TT. Scale bars, 200 nm. The arrowheads indicate the polymer coatings. (**B**) Hydrodynamic size distribution profiles and (**C**) zeta potential of HRA and HRA@D-TT (*n* = 3). (**D**) X-ray photoelectron spectroscopy (XPS) C1s and N1s spectra of HNI and HNI@TT. (**E**) FTIR spectra of HNI, HNI@TT, DOX, and HNI@D-TT. a.u., arbitrary unit. (**F**) Fluorescence spectra of DOX, HNI@TT, and HNI@D-TT. (**G**) Schematic illustration of reversible polymer shell variation in response to pH changes. The variation in (**H**) particle size and (**I**) zeta potential of HNI@D-TT and HNI@D-TA in different pH (*n* = 3). (**J**) Representative FRET images of 4T1 cells incubated with HNI-(DiI + DiO)@TT under normoxic or hypoxic conditions. The fluorescence intensities were analyzed following the white lines on the cells. Scale bars, 20 μm. DiO, excitation wavelength (*E*_x_) = 488 nm and emission wavelength (*E*_m_) = 505 to 550 nm; DiI, *E*_x_ = 549 nm and *E*_m_ = 560 to 615 nm. (**K**) The quantification analyses of FRET ratio through ImageJ (*n* = 3). (**L**) The ultraviolet absorption spectra of HNI micelles with/without Na_2_S_2_O_4_ treatment in PBS for 8 hours. (**M**) In vitro DOX release from HRA@D-TT and HRA@D-TA in different pH buffers (*n* = 3). (**N**) In vitro ATRA release from HRA@D-TT in different pH buffers with hypoxia or normoxia (*n* = 3). Data are expressed as means ± SD, and the significance was analyzed using two-tailed Student’s *t* test. ***P < 0.001. h, hours.

The transmission electron microscopy (TEM) revealed that HRA@D-TT exhibited a classical core-shell morphology with a shell thickness of ~25 nm, distinct from the sphericity of the HRA (Fig. 2A). This structural transition was also evidenced via the dynamic light scattering analysis, which indicated an increase in particle size and zeta potential after coating (Fig. 2, B and C, and table S1). The observed rise in surface potential was attributed to the introduction of numerous amino groups and the formation of quaternary ammonium groups under neutral conditions.

To further investigate the nanocore encapsulation by the functional shell and the DOX loading mechanism, control groups HNI@TT (with no drug) and HNI@D-TT (with DOX only) were prepared using the same method. X-ray photoelectron spectroscopy (XPS) exhibited enhanced C–O and C=O responses and an increased proportion of C=N–C following shell encapsulation (Fig. 2D). This suggested the presence of phenolic hydroxyl groups and Schiff base on the surface of HNI@TT, caused by TA-TEPA coating. As seen in Fig. 2E, Fourier transform infrared (FTIR) analysis demonstrated a hypochromic shift from 3415 and 1742 cm^−1^ of HNI toward 3390 and 1694 cm^−1^ of HNI@TT, respectively, indicating the formation of hydrogen bonds during the attachment of TA-TEPA shell to nanocore. Additionally, new absorption signals at 1590, 1500, and 1347 cm^−1^ were noted in the spectrum of HNI@TT, characteristic of the aromatic rings of TA, while the new peak at 759 cm^−1^ could be ascribed to the bending vibration of C–H in benzene rings. The spectra of HNI@D-TT further showed a peak at 1285 cm^−1^, which corresponded to the C–O stretching vibration in DOX, confirming the successful drug loading (DL). Moreover, the peaks of DOX blue-shifted to 3367 and 1678 cm^−1^ in HNI@D-TT, indicating the presence of hydrogen bonds between DOX and TA-TEPA shell. The abundance of aromatic rings in drugs and carriers implies that π–π stacking interaction may be a crucial driving force in the assembly process. This hypothesis was supported by fluorescence spectra (Fig. 2F) and ultraviolet spectra (fig. S8), which showed a fluorescence quenching effect and a red shift in the maximum ultraviolet absorption wavelength of DOX after loading the drug, respectively.

### The stimuli-responsive capacity and drug release behavior

To extend the intercellular transport lifetime and enhance persistent toxicity, TEPA was used as a cross-linker, allowing the nanoplatform to respond repeatedly to pH fluctuations (Fig. 2G). The underlying mechanisms of this reversible variation in the polymer shell’s structure were as follows: (i) the –C=N– bond dissociated under acidic conditions, loosening the shell, while the TEPA cross-linking prevented TA shedding and allowed the shell to redensify under neutral conditions; and (ii) TEPA underwent protonation at pH 5.0, inducing electrostatic repulsion, and was deprotonated at pH 7.4. To further investigate the contribution of TEPA, the HRA@D-TA was fabricated as a control through the oxidation-induced oligomerization of TA. Both HRA@D-TA and HRA@D-TT exhibited high stability under storage ambient conditions (fig. S9). In contrast to the TA monolayer, HRA@D-TT showed a reversible size change from 160 nm at pH 7.4 to nearly 300 nm at pH 5.0, with the surface potential performing heightened sensitivity to the increased acidity (Fig. 2, H and I).

Subsequently, the fluorescence resonance energy transfer (FRET) was used to evaluate the hypoxia-triggered cargo release. The nanoplatform with inner encapsulating the FRET pair, DiO and DiI, was designed to facilitate the visualization of the release process [HNI-(DiI + DiO)@TT]. As depicted in Fig. 2J, a distinct FRET signal was detected in 4T1 cells under oxygen-deprived conditions, characterized by elevated green fluorescence from DiO and quenched red fluorescence from DiI. The FRET ratio, defined as the ratio of DiI to DiO fluorescence intensities, also showed a marked decline, demonstrating the rapid disassembly of micelles in response to hypoxia (Fig. 2K). The same conclusion could also be drawn from ultraviolet spectra analysis, which identified the nitroimidazole group as the hypoxia-sensitive site, leading to the reductive conversion to the hydrophilic aminoimidazole group (Fig. 2L).

Subsequently, the drug release profiles of HRA@D-TT were investigated. As depicted in Fig. 2M, HRA@D-TT exhibited minimal DOX release at physiological pH 7.4, indicative of its potential to mitigate systemic toxicity arising from premature “burst release.” Upon exposure to an acidic environment (pH 5.0), a marked acceleration in DOX release was observed, increasing approximately threefold. For comparative purposes, the release behavior of HRA@D-TA was also evaluated under varying pH conditions. At neutral pH, HRA@D-TA displayed premature drug leakage. This was likely attributed to the inherently looser structure of the pure TA shell. Unlike TA-TEPA, this shell failed to achieve dense encapsulation of DOX. When the pH of the release medium was lowered from 7.4 to 5.0, HRA@D-TA exhibited markedly accelerated and substantial drug release, with its 24-hour cumulative release surpassing that of HRA@D-TT. However, such rapid release was achieved at the cost of the structural disintegration of the HRA@D-TA outer shell (fig. S10). This disintegration was ascribed to the protonation of TA’s phenolic hydroxyl groups within the acidic environment, a process that destabilized the polymeric network and triggered its dissociation. Consequently, the compromised shell was unable to reestablish effective DOX encapsulation even upon restoration of neutral pH conditions.

To further investigate the influence of recyclable response characteristics on drug release behavior, HRA@D-TT was subjected to alternating incubation at pH 7.4 and 5.0 for 6-hour intervals. This protocol was designed to simulate the pH variations encountered during lysosomal-cytoplasmic transport in cellular environments. The incubation cycle was repeated seven times, with the amount of released DOX measured after each cycle. As shown in fig. S11, HRA@D-TT demonstrated sustained responsiveness to environmental pH changes, maintaining continuous DOX release until the sixth incubation cycle. In contrast, HRA@D-TA exhibited diminished release capacity, with only 5% DOX release detected during the fourth incubation cycle. These findings demonstrated that HRA@D-TT effectively prevented premature drug release under neutral pH conditions while maintaining sustained release capacity in acidic environments.

Moreover, HRA@D-TT had superior hypoxia sensitivity, facilitating rapid ATRA release when exposed to a low-oxygen environment. As illustrated in Fig. 2N, the release of ATRA under the combined stimuli of low oxygen and high acidity significantly exceeded that initiated by hypoxia alone, suggesting that the structure of the polymer shell constrained the ATRA release from the inner core. The enhanced release could be attributed to the surface loosening at low pH, which endowed the nanoplatform with a superior capacity for rapid drug release under certain conditions.

### Intracellular tracking and cytotoxicity assessment

**Fig. 3.**
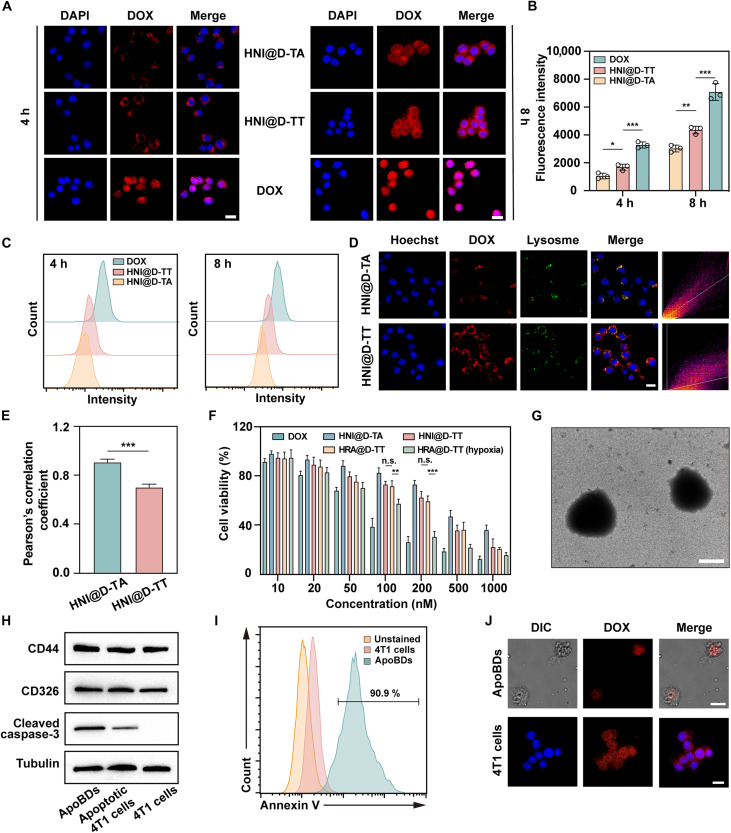
The intracellular tracking, cytotoxicity, and ApoBDs characterization. (**A**) Confocal images and (**B**) flow cytometry quantification of 4T1 cells treated with DOX, HNI@D-TT, and HNI@D-TA for 4 and 8 hours. Scale bar, 20 μm. (**C**) Representative flow cytometry images of 4T1 cells internalized various formulations. (**D**) Lysosome escape behavior of HNI@D-TT and HNI@D-TA and (**E**) the corresponding Pearson’s correlation coefficients in 4T1 cells (*n* = 3). Scale bar, 20 μm. (**F**) The cytotoxicity against 4T1 cells after various treatments for 48 hours (*n* = 3). (**G**) TEM image of ApoBDs. Scale bar, 1 μm. (**H**) Western blot analysis of ApoBDs and apoptotic cells. (**I**) Flow cytometry characterization of annexin V^+^ ratio of ApoBDs. (**J**) Confocal images of ApoBDs and 4T1 cells treated with ApoBDs. Scale bars, 1 μm and 20 μm. DIC, differential interference contrast. Data are expressed as means ± SD. The significance in (E) was calculated through two-tailed Student’s *t* test. The significance analysis in (B) was carried out through one-way analysis of variance (ANOVA). *P < 0.05; **P < 0.01; ***P < 0.001; n.s., not significant. h, hours.

Efficient cellular uptake is acknowledged as a critical prerequisite for the therapeutic effectiveness of nanomedicines. To assay the internalization efficiency, 4T1 cells were incubated with DOX solution, HNI@D-TA, and HNI@D-TT for 4 and 8 hours. The autofluorescence signal of DOX was subsequently detected using a confocal laser scanning microscope (CLSM; Fig. 3A) and further quantitated by flow cytometry (Fig. 3, B and C). Cells treated with DOX solution exhibited accelerated fluorescence colocalization within the nucleus, accompanied by the highest intensity of red fluorescence. This observation can be attributed to the absence of an effective regulatory mechanism in the solution formulation, which permitted the rapid influx of free DOX into the cells and subsequent interaction with the nucleus. Conversely, HNI@D-TT and HNI@D-TA primarily displayed a punctate distribution in the cytoplasm during the initial phase of uptake, suggesting internalization via endocytosis. As the uptake process progressed, the fluorescence of DOX in the nanoparticle-treated groups gradually colocalized with the nucleus. Notably, HNI@D-TT achieved an internalization efficiency 1.5 times higher than the TA-covered counterpart, possibly due to its higher surface potential (*43*). Additionally, the presence of catechol and amine groups was also reported to collectively enhance the cellular uptake (*44*).

While rapid tumor cell endocytosis is crucial for nanodrug efficacy, it is merely the initial step. Timely lysosome escape could reduce the retention of weakly basic drugs due to ion trapping in acid lysosomal conditions, thereby enhancing cytotoxicity (*45*). As depicted in Fig. 3 (D and E), most of the red DOX fluorescence in cells treated with HNI@D-TA overlapped with LysoTracker green–stained lysosomes, whereas this colocalization signal was diminished in the HNI@D-TT treatment group. These results indicated that HNI@D-TT can diffuse more rapidly into the cytoplasm following internalization, facilitated by a proton sponge effect from unprotonated amino groups in TEPA, as previously reported.

The in vitro cytotoxicity of HRA@D-TT was examined through a 3-(4,5-dimethylthiazol-2-*yl*)-2,5-diphenyltetrazolium bromide (MTT) assay. As seen in Fig. 3F, the cell viability inhibitory followed the rank order of DOX > HNI@D-TT > HNI@D-TA, agreeing with the profiles of cellular uptake and lysosomal escape efficiency. Notably, when cultured in a normoxic condition, cells treated with HRA@D-TT showed negligible difference in viability compared to those treated with HNI@D-TT, with similar median inhibitory concentration (IC_50_) values of 277.9 and 253.6 nM, respectively. In comparison, exposure to hypoxia significantly enhanced the cytotoxicity in HRA@D-TT, evidenced by a reduced IC_50_ of 116.7 nM. This efficacy discrepancy resulting from the divergent incubation environment further confirmed that HNI micelles disintegrated under hypoxic conditions, releasing ATRA and exerting a synergistic antitumor advantage. Additionally, the carriers HNI@TT and HNI@TA demonstrated excellent biocompatibility with limited cytotoxicity even at a concentration as high as 100 μg/ml (fig. S12).

### Enhanced intercellular permeation via ApoBDs

DOX-induced apoptosis initiated the generation of ApoBDs, which varied in size. To explore the neighboring effect mediated by ApoBDs, we first purified them from the cell supernatants with HNI@D-TT induction through a gradient centrifugation protocol (*46*). As seen in Fig. 3G, TEM confirmed that ApoBDs were spherical vesicles, measuring ~1.5 μm in diameter. Furthermore, ApoBDs were comparable to the 4T1 cells in the expression of membrane markers CD44 and CD326 but displayed elevated levels of cleaved caspase-3 owing to the high degree of apoptosis (Fig. 3H). Annexin V was applied to label the externalized phosphatidylserine in apoptotic cells, and, as shown in Fig. 3I, 90.9% of the isolated ApoBDs exhibited a high annexin V^+^ ratio, confirming their high purity. Additionally, DOX fluorescence was detected in the isolated ApoBDs, which could be internalized by 4T1 cells, indicating their role as a reservoir for transporting remaining drugs (Fig. 3J).

**Fig. 4.**
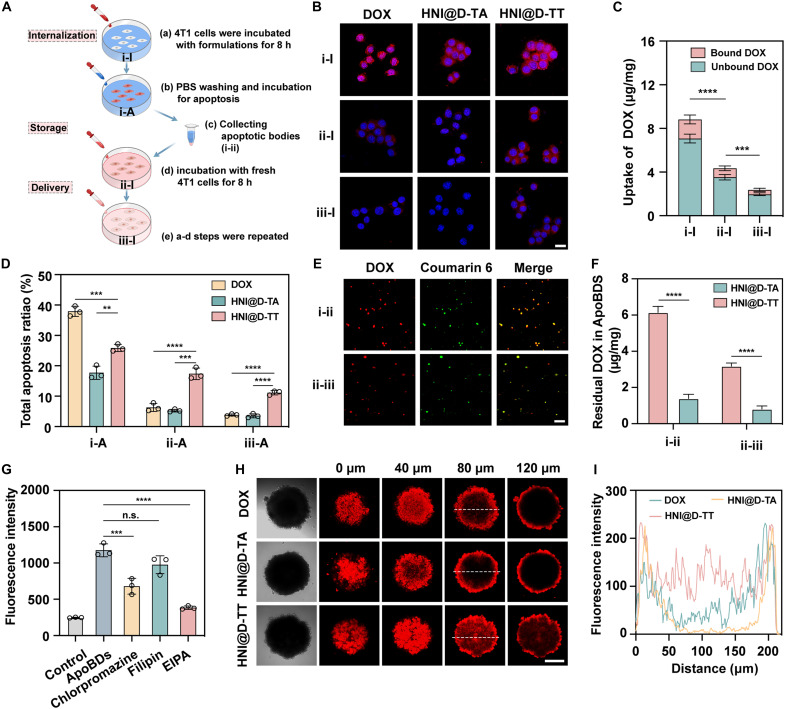
The high penetration of HNI@D-TT mediated by ApoBDs. (**A**) Schematic illustration for investigating the intercellular delivery of HNI@D-TT. (**B**) The fluorescence images of 4T1 cells after exposure to various treatments. Scale bar, 20 μm. (**C**) The amounts of bound and unbound DOX in cells (i-A), (ii-A), and (iii-A) treated with HNI@D-TT (*n* = 3). (**D**) Apoptosis rates of 4T1 cells after exposure to various treatments (*n* = 3). (**E**) The fluorescence images of ApoBDs (i-ii) and (ii-iii) isolated from cell supernatants after treatment with coumarin 6–labeled HNI@D-TT. Scale bar, 5 μm. (**F**) The residual amounts of DOX in ApoBDs (i-ii) and (ii-iii) after the treatment of HNI@D-TT and HNI@D-TA (*n* = 3). (**G**) The cellular uptake of ApoBDs after pre-treatment with different endocytosis inhibitors (*n* = 3). (**H**) The penetration efficiency of DOX, HNI@D-TT, and HNI@D-TA in the 3D tumorsphere model and (**I**) corresponding quantitative results. Scale bar, 100 μm. Data are expressed as means ± SD and statistical significance was carried out through one-way ANOVA. **P < 0.01; ***P < 0.001; ****P < 0.0001; n.s., not significant. h, hours.

Our hypothesis proposed that, after the initial drug endocytosis, HNI@D-TT with residual drugs was stored in ApoBDs and subsequently enhanced therapeutic efficiency through ApoBD-mediated intercellular transfer. To test this hypothesis, 4T1 cells in well (i) were exposed to various formulations for 8 hours, after which the supernatants were replaced with phosphate-buffered saline (PBS) to facilitate further apoptosis. Subsequently, ApoBDs were isolated and incubated with fresh cells in well (ii), with this procedure repeated three times (Fig. 4A). From CLSM observation, the cells incubated with DOX solution or HNI@D-TA showed weak red fluorescence in the second cycle, which became inviable in the third cycle, suggesting their limited delivery capacity to the neighboring cells. In contrast, despite a gradual decrease in fluorescence intensity, cells treated with HNI@D-TT in well (iii) still exhibited a distinct signal, attributed to the controllable pulsatile release of DOX, which facilitated the retention of the remaining drugs (Fig. 4B). Quantitative analysis revealed that 15 to 20% of DOX entered the nucleus in cells treated with HNI@D-TT, due to strong binding affinity with topoisomerase II, while the remaining drugs remained active (Fig. 4C). Moreover, cells from each treatment round were collected to assess apoptotic ratio using flow cytometry. As shown in Fig. 4D and fig. S13, cells treated with HNI@D-TT still exhibited an 11% apoptotic rate during the final incubation, demonstrating the satisfactory penetration effect. Although the DOX solution initially induced extensive apoptosis, its excessive consumption substantially impaired the effectiveness of subsequent treatments.

The fluorescence of coumarin 6 encapsulated in HNI micelles within ApoBDs was also detected. As illustrated in Fig. 4E, the overlapped fluorescence signals between red and green confirmed the coencapsulation of DOX and coumarin 6 in ApoBDs. However, with the continued intercellular transport, the consumption of DOX led to a reduction in red fluorescence within ApoBDs (ii-iii), while the fluorescence of coumarin 6 remained stable. This illustrated that the inert behavior of HNI micelles under normoxic conditions prevented cargo leakage. Additionally, a higher accumulation of unbound drugs was measured within ApoBDs (i-ii) and (ii-iii) in the HNI@D-TT group, indicating that the HNI@D-TT–mediated pulsatile release enhanced the neighboring effect by increasing the proportion of active drugs within ApoBDs (Fig. 4F). Furthermore, the cellular internalization mechanism of ApoBDs was investigated by using diverse endocytosis inhibitors, including chlorpromazine, filipin, and 5-(Ethylisopropyl)amiloride (EIPA), which prevent clathrin-, caveolin-, and macropinocytosis-mediated endocytosis, respectively (Fig. 4G). Among these, EIPA showed the most significant effect, highlighting the predominant role of macropinocytosis in ApoBDs internalization. Chlorpromazine also decreased the fluorescence intensity of DOX in treated cells, indicating the involvement of clathrin-mediated endocytosis in the cell uptake. Given that endo/lysosomes serve as key sites for cargo sorting during macropinocytosis and clathrin-mediated endocytosis, HNI@D-TT stored in ApoBDs could facilitate drug release triggered by pH variations upon internalization into cells.

Next, we explored the distribution of HNI@D-TT within a three-dimensional (3D) tumor sphere model, which was established to simulate drug penetration in tumors. As depicted in Fig. 4 (H and I), the group treated with HNI@D-TT displayed the most homogeneous drug distribution and superior penetration behavior, reaching a depth of 80 μm into the center of the tumor sphere. Contrastively, the red fluorescence of the free DOX and HNI@D-TA group was confined to the periphery of the tumor spheroid, indicating suboptimal penetration compared to the HNI@D-TT treatment. Overall, the lower internalization and cytotoxicity of HNI@D-TA resulted in limited penetration depth within spheres, and its nanoparticle size also proved disadvantageous for penetration compared to free DOX (*45*). Conversely, HNI@D-TT leveraged pulsatile drug release to consistently elicit extensive apoptosis, enhancing penetration through the ApoBD-mediated neighboring effect.

### In vitro regulation of characteristics associated with stemness

**Fig. 5.**
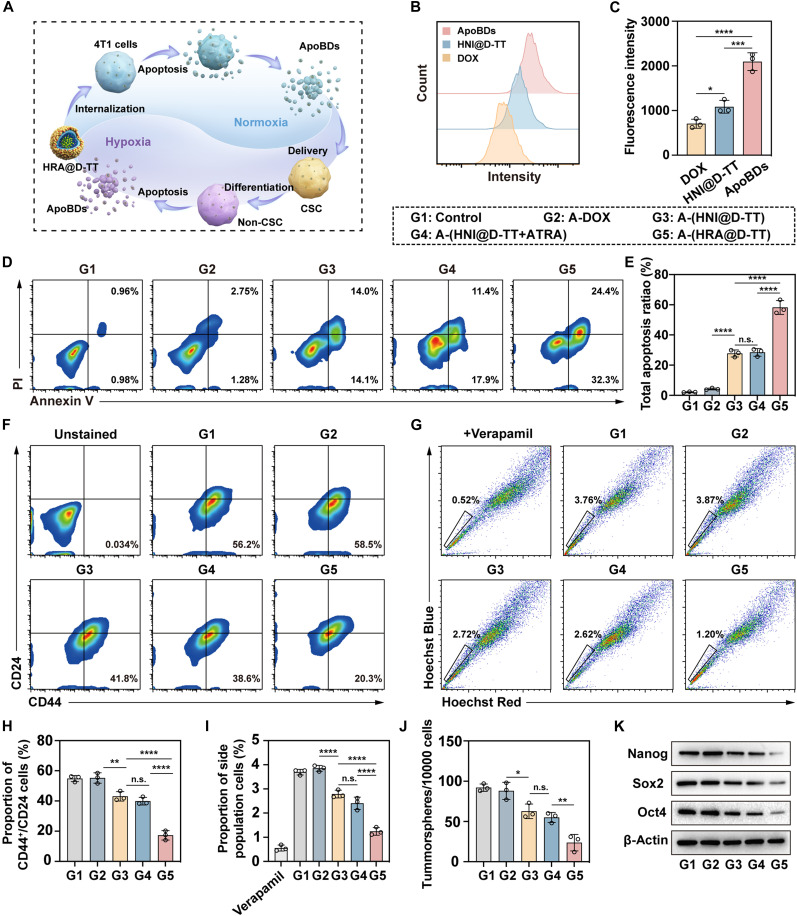
In vitro cancer stemness suppression capacity of HRA@D-TT. (**A**) Illustration of the incubating method for evaluating the anti-CSC ability of HRA@D-TT. 4T1 cells were incubated with HRA@D-TT for 8 hours under normoxic conditions and further incubated in the fresh medium for 12 hours to induce apoptosis. Next, ApoBDs were isolated and added to the tumorsphere cells’ medium under hypoxia. (**B**) Flow cytometry measurement and (**C**) fluorescence quantification of tumorsphere cells treated with DOX, HNI@D-TT, and ApoBDs (*n* = 3). (**D**) Apoptosis assay and (**E**) quantification of BCSCs after various ApoBD treatments (*n* = 3). Representative flow cytometry images of relative percentages of (**F**) CD44^+^/CD24^−^ cells in tumorsphere cells and (**G**) SP cells in 4T1 cells after various treatments. (**H** and **I**) Quantification analysis in (F) and (G) (*n* = 3). (**J**) The tumorsphere formation assay of 4T1 cells after various treatments for 7 days. The number of the resultant tumor spheres was quantified (*n* = 3). (**K**) Relative protein levels of Nanog, Oct4, and Sox2 in treated 4T1 cells. Data are represented as means ± SD and statistical significance was carried out through one-way ANOVA. *P < 0.05; **P < 0.01; ***P < 0.001; ****P < 0.0001; n.s., not significant.

As previously described, the outer shell has been demonstrated to enable DOX to effectively access deep tumor sites. We subsequently examined the efficacy of ATRA encapsulated within the platform in differentiating deep CSCs (Fig. 5A). Initially, we established the CSC-rich 3D tumor model from the 4T1 breast cancer cells, which is recognized as a reliable platform for evaluating stemness properties (*47*). After three passages in serum-free and low-adhesion culture systems, the spheroid-shaped breast CSCs (BCSCs) were analyzed by flow cytometry for CD44 and CD24 expression. As depicted in fig. S14A, 56.0% of tumorsphere cells displayed high levels of CD44^+^/CD24^−^ expression. The stem-related proteins, including Sox2, Nanog, and Oct4, were also significantly up-regulated in BCSCs, setting the stage for subsequent validation experiments (fig. S14B).

The endocytosis efficiency of drug-loaded ApoBDs is essential for targeting cells with stem-like phenotypes. To verify this, BCSCs were subjected to various treatments, including DOX solution, HNI@D-TT, and the ApoBDs (from HNI@D-TT–induced apoptosis) under hypoxic conditions for 4 hours. As illustrated in fig. S15, the ApoBD treatment resulted in the strongest DOX fluorescence, whereas the DOX solution alone exhibited the weakest. Flow cytometry analysis also revealed that the uptake of ApoBDs by BCSCs was threefold higher compared to the DOX solution and 1.9 times higher than HNI@D-TT (Fig. 5, B and C). This enhanced cellular uptake may be attributed to two principal mechanisms. First, the elevated metabolic demands of CSCs enhance their capacity to internalize potential nutrient sources from the surrounding microenvironment, notably including ApoBDs. Furthermore, the membrane architecture of ApoBDs can modulate the physicochemical properties of the cellular membrane interface, thereby impairing the functional activity of drug efflux transporters to a certain extent. Atomic force microscopy (AFM) analysis of BCSCs following various treatments revealed that drug-containing ApoBDs significantly increased cell surface roughness (Ra), disrupting the normal cell membrane microenvironment (fig. S16A). This structural alteration appeared to correlate with reduced expression of p-glycoprotein (P-gp), a membrane efflux transporter responsible for drug resistance in cancer cells (fig. S16B) (*48*). These findings suggested that ApoBD-mediated changes to membrane architecture may compromise the drug efflux mechanisms, potentially enhancing therapeutic efficacy by preventing P-gp–mediated expulsion of chemotherapeutic agents from the cells.

In subsequent experiments, BCSCs were incubated under hypoxic conditions for 48 hours with ApoBDs derived from different treatment groups. These ApoBDs were collected from culture supernatants of cells treated with various formulations and were designated as follows: A-DOX (from DOX-treated cells), A-(HNI@D-TT) (from HNI@D-TT–treated cells), A-(HNI@D-TT + ATRA) (from HNI@D-TT + ATRA–treated cells), and A-(HRA@D-TT) (from HRA@D-TT–treated cells). Flow cytometry to assess the apoptotic rate of CSCs showed that the A-(HRA@D-TT) group displayed superior anti-CSC efficacy among all treatments (Fig. 5, D and E). Notably, even in the absence of differentiation inducers, HNI@D-TT demonstrated the ability to inhibit the survival of CSCs mediated by ApoBDs, which can be attributed to the cytotoxic effect of residual DOX retained within the nanoplatform. Compared with HRA@D-TT, supplementation with free ATRA exhibited a weaker effect, and the induced apoptosis rate showed no significant difference from that of HNI@D-TT. This phenomenon suggested that free ATRA exhibited insufficient efficacy against CSCs in hypoxic microenvironments due to its premature depletion in normoxic regions. In contrast, the protective effect of the HNI nanocore enabled spatiotemporally specific release of ATRA at hypoxic sites. This sequential release capability enhanced the efficiency of ATRA in inducing CSC differentiation and, in combination with DOX, achieved comprehensive tumor killing. The Calcein AM/PI double staining further confirmed that HRA@D-TT can use ApoBDs to enhance the killing effect on deep-seated CSCs (fig. S17). Additionally, we examined changes in the CD44^+^/CD24^−^ ratio in BCSCs following treatments. A significant reduction in cells with this phenotype was measured in the A-(HRA@D-TT) group, demonstrating the considerable potential of HRA@D-TT for inducing differentiation and reducing the proportion of malignant cells (Fig. 5, F and H). Side population (SP) cells are acknowledged as a heterogeneous group, which were identified from 4T1 cells by classical Hoechst 33342 staining (*49*). As seen in Fig. 5 (G and I), the proportion of SP cells was reduced from 3.76 to 1.20% by the treatment with ApoBDs containing HRA@D-TT, slightly higher than the 0.52% reduction achieved by ABC transporter inhibitor verapamil. Tumor sphere formation assay and Western blot analysis were then conducted to evaluate the self-renewal and differentiation capabilities of BCSCs. Significant reductions of tumorspheres were observed in 4T1 cells after 7 days of treatments (Fig. 5J and fig. S18), along with the decreased levels of Sox2, Oct4, and Nanog protein (Fig. 5K). Overall, these results strongly demonstrated the synergistic benefits of cascade tumor penetration and differentiation in eliminating CSCs.

### In vivo biodistribution and enhanced penetration

**Fig. 6.**
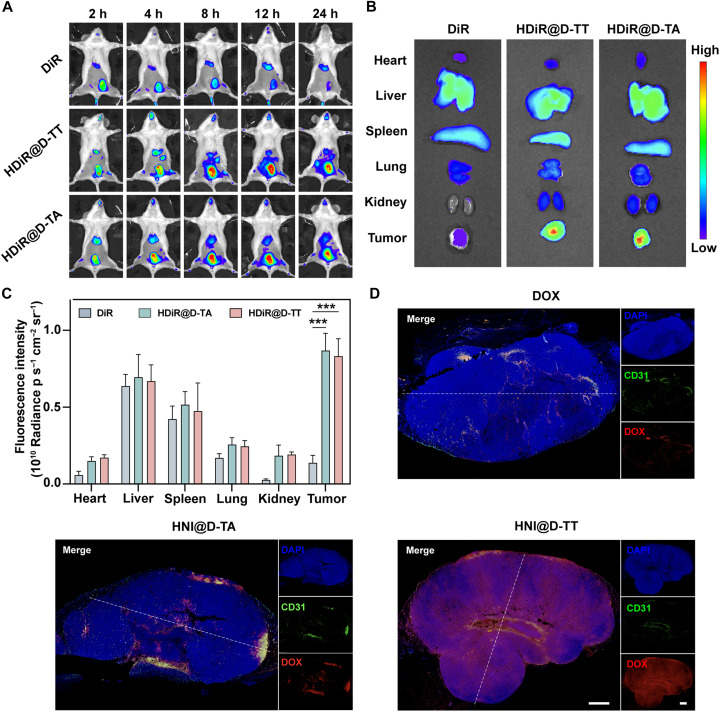
In vivo biodistribution and tumor penetration of nanoplatforms. (**A**) Fluorescence images of 4T1 tumor-bearing mice intravenously administrated with DiR, HDiR@D-TT, and HDiR@D-TA at predetermined time points (2, 4, 8, 12, and 24 hours). (**B**) Fluorescence images and (**C**) semiquantitative results of main organs and tumors after intravenous injection at 24 hours (*n* = 3 mice). (**D**) CLSM images of tumor sections after intravenous injection of DiR, HDiR@D-TT, and HDiR@D-TA at 24 hours. Hoechst 33342, blue; DOX, red; CD31, green. Scale bar, 1 mm. Data are represented as means ± SD and statistical significance was carried out through one-way ANOVA. ***P < 0.001. h, hours.

To further understand the nano-bio fate of the nanoplatform in living organisms, HNI@D-TT was labeled with DiR (HDiR@D-TT) to monitor in vivo behaviors. As depicted in Fig. 6A, a prominent fluorescence signal persisted at the tumor site in the mice treated with HDiR@D-TT and HDiR@D-TA up to 24 hours post–intravenous injection, contrasting with the diminished intensity with DiR solution due to its rapid elimination. Moreover, tumors and main organs were harvested from mice and imaged at 24 hours postinjection for quantitative biodistributions. As illustrated in Fig. 6 (B and C), the mice administered with HDiR@D-TT and HDiR@D-TA exhibited significantly higher fluorescence intensities at the tumor sites compared to normal organs, due to the enhanced permeability and retention (EPR) effect inherent in their nanostructures. Additionally, the intratumoral distribution of HNI@D-TT was further validated in a 4T1-bearing mouse model. Here, green CD31 staining marked the vasculature, and red indicated the distribution of DOX. As depicted in Fig. 6D and fig. S19, HNI@D-TT amplified the neighboring effect, overcoming permeability barriers and achieving optimal penetration efficiency. Although HNI@D-TA displayed similar tumor accumulation to HNI@D-TT, its distribution was confined to the perivascular areas, consistent with the in vitro tumor spheroid penetration study results. The DOX solution exhibited the poorest tumor permeability, attributable to its inferior tumor targeting and accelerated metabolism.

To evaluate the capacity of hypoxia-responsive nanocores to enhance intratumoral drug distribution, we conducted comparative studies using DiR solution, DiR + HNI@D-TT, and HDiR@D-TT administered intravenously to tumor-bearing mice. Hypoxic regions within tumor tissues were identified through HIF-1α immunostaining, visualized as green fluorescence. As demonstrated in fig. S20, CLSM analysis revealed that free DiR (red fluorescence) exhibited minimal colocalization with HIF-1α–positive hypoxic regions. This pattern indicated that unformulated DiR predominantly accumulated in well-vascularized, normoxic tumor areas while achieving limited penetration into hypoxic regions. In contrast, the hypoxia-responsive nanocore formulation (HDiR@D-TT) demonstrated marked colocalization between DiR and HIF-1α signals, confirming preferential accumulation within hypoxic tumor compartments. These findings confirmed that the hypoxia-responsive nanocore enabled spatially controlled drug release specifically within hypoxic tumor regions, thereby enhancing the efficacy of differentiation therapy.

### In vivo therapeutic effect and anti-CSC study

**Fig. 7.**
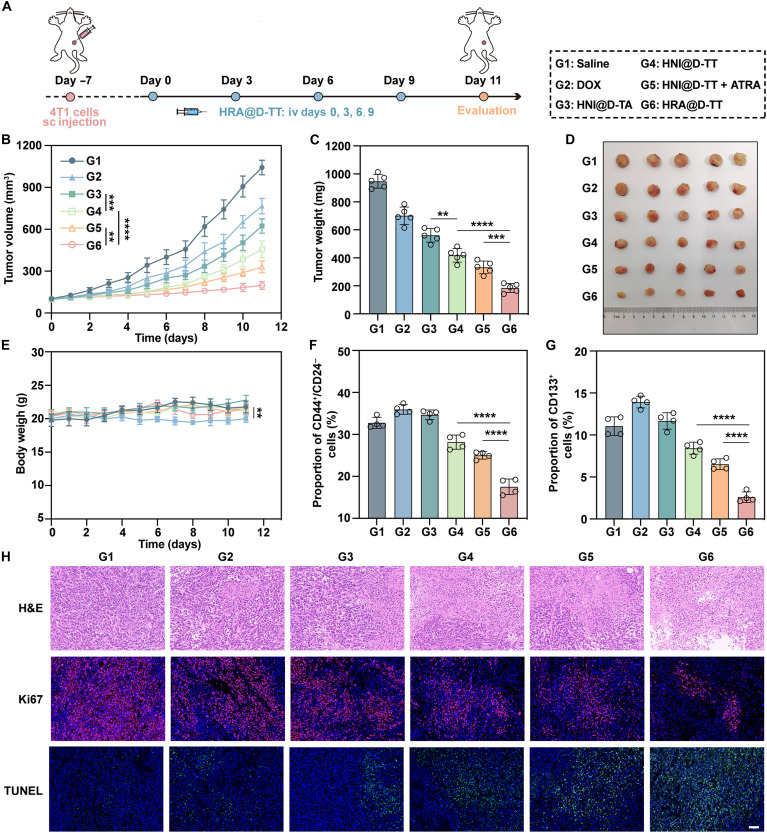
In vivo antitumor efficiency assessment of HRA@D-TT. (**A**) Schema depicting the 4T1 tumor-bearing model construction and treatment schedule. sc, subcutaneous; iv, intravenous. (**B**) Average tumor growth profiles after different treatments (*n* = 5 mice). (**C**) Average tumor weight and (**D**) tumor photos at the experimental endpoint (*n* = 5 mice). (**E**) Body weight changes of mice during treatment (*n* = 5 mice). (**F**) The relative proportion of CD44^+^/CD24^−^ cells in tumor tissues (*n* = 4 mice). (**G**) Qualification of CD133^+^ cells in tumor tissues (*n* = 4 mice). (**H**) Representative H&E, Ki67, and TUNEL staining images of tumor sections after different treatments. Scale bar, 100 μm. Data are represented as means ± SD and statistical significance was analyzed through one-way ANOVA. **P < 0.01; ***P < 0.001; ****P < 0.0001.

The favorable tumor accumulation and penetration capacities of the sequential drug release nanoplatform promoted us to further investigate the efficacy of HRA@D-TT in controlling tumor progression using the 4T1 breast tumor model. Orthotopic tumors were treated four times with various formulations, including saline, DOX, HNI@D-TA, HNI@D-TT, HNI@D-TT + ATRA, and HRA@D-TT, at a DOX-equivalent dose of 2 mg/kg, before the mice were euthanized (Fig. 7A). As shown in Fig. 7 (B to D), DOX solution and HNI@D-TA exhibited the modest antitumor effects, possibly limited by poor tumor accumulation and inadequate cytotoxicity, respectively. HNI@D-TT, benefiting from its superior permeability, significantly inhibited tumor growth, but the tumor volume still reached ~500 mm^3^. Notably, in contrast to the physical mixture of HNI@D-TT and ATRA, HRA@D-TT fully harnessed synergistic effects, achieving maximal tumor suppression. Despite administering low doses of free DOX, mice experienced weight loss over time (Fig. 7E). Conversely, the HRA@D-TT–treated and saline-treated groups showed similar results in terms of body weight, hematoxylin and eosin (H&E) staining of major organs, and liver and kidney function tests (figs. S21 and S22), indicating the excellent biosafety of the nanoplatform and the ability to mitigate the systemic toxicity of DOX.

Given its significant tumor inhibitory capacity, we subsequently investigated the effectiveness of HRA@D-TT in eradicating stem-related phenotypes. Tumors harvested from mice were subjected to multiple flow cytometry strategies to evaluate the proportions of CSCs, specifically CD133^+^ and CD44^+^/CD24^−^ populations. As shown in Fig. 7 (F and G) and fig. S23, chemotherapy alone risked enriching CSCs and enhancing drug resistance. However, HNI@D-TT enhanced drug accumulation in CSCs via ApoBDs and countered their evolution. Both analytical approaches demonstrated that HRA@D-TT effectively promoted differentiation and reduced the CSC population. In contrast, mixtures of HNI@D-TT and ATRA without encapsulation by the protective nanocore showed limited efficacy. Moreover, immunofluorescence staining of key stemness markers (Sox2, Oct4, and Nanog) revealed significantly reduced fluorescence in the HRA@D-TT group, highlighting its effectiveness in eliminating CSCs (fig. S24). H&E staining further indicated that HRA@D-TT induced the most substantial damage to tumor tissue (Fig. 7H). Additionally, HRA@D-TT treatment resulted in heightened levels of tumor apoptosis and inhibited tumor cell proliferation, as evidenced by Ki67 and terminal deoxynucleotidyl transferase–mediated deoxyuridine triphosphate nick end labeling (TUNEL) staining. Collectively, these results indicated that HRA@D-TT combines superior tumor penetration with spatially dependent CSC elimination capabilities, which are capable of inhibiting tumor growth through a dual-pronged approach.

### Therapeutic applicability of HRA@D-TT in diverse tumor models

To evaluate the generality of the cascade permeation enhancement and differentiation strategy embodied by HRA@D-TT, its antitumor activity was further investigated in a CT26 murine colon carcinoma model (fig. S25A). Following 12 days of treatment, HRA@D-TT exhibited superior tumor growth suppression, achieving a tumor inhibition rate of 75.6% and significantly inhibiting tumor progression compared to other treatment groups. Furthermore, HNI@D-TT, lacking the differentiation agent ATRA within the hypoxic core, still demonstrated an inhibition rate of ~48%, surpassing the effects observed with monotherapy using DOX solution or HNI@D-TA (fig. S25, B to E). As shown in fig. S25 (F and G), histopathological analysis of HRA@D-TT–treated tumors revealed increased TUNEL-positive signals alongside reduced Ki67 expression.

Building upon the demonstrated efficacy in subcutaneous 4T1 and CT26 tumor models, we further investigated the therapeutic potential of this approach against a highly invasive orthotopic pancreatic tumor model (fig. S26A). This model was characterized by inherent physical and physiological barriers that impede drug perfusion, contributing to chemoresistance (*50*). Moreover, a CSC-enriched niche significantly promotes pancreatic cancer cell invasion and growth (*51*). As shown in fig. 26 (B to D), the mice treated with DOX solution exhibited invasive tumor growth, whereas HNI@D-TT delayed tumor progression. Crucially, HRA@D-TT, incorporating spatially controlled release of ATRA, achieved optimal tumor suppression, evidenced by a significant reduction in bioluminescence signal and prolonged survival. Immunofluorescence staining of tumor sections further demonstrated that HRA@D-TT treatment resulted in minimal CD133-positive signal, indicating that the combined propenetration and differentiation therapy effectively targeted CSCs (fig. S26E). Collectively, these findings demonstrated that HRA@D-TT offered distinct therapeutic advantages and broad-spectrum efficacy across diverse tumor models.

### Therapeutic advantages of HRA@D-TT in overcoming multidrug resistance

The enhancement of both intrinsic and acquired drug resistance in tumor cells frequently correlates with an increased proportion of CSCs. SP cells, characterized by high expression of drug efflux proteins, represent a well-established model of chemoresistant cells. We isolated this population (4T1-SP) from parental 4T1 cells using Hoechst 33342–based sorting and maintained them in serum-free suspension culture for 7 days (fig. S27A). As illustrated in fig. S27 (B and C), the suspended 4T1-SP cells formed characteristic tumor spheroids and exhibited a high proportion of CD44^+^/CD24^−^ cells, confirming CSC enrichment. Notably, 4T1-SP cells demonstrated enhanced intrinsic drug resistance compared to parental 4T1 cells, as evidenced by their significantly lower sensitivity to DOX [IC_50 (4T1-SP)_ = 870 nM and IC_50 (4T1)_ = 80.47 nM]. However, under simulated hypoxic conditions, HRA@D-TT treatment markedly enhanced their DOX sensitivity (IC_50_ = 362.9 nM), indicating effective synergistic cytotoxicity against resistant cells (fig. S27D). To evaluate HRA@D-TT’s differentiation effects on CSCs within hypoxic tumor regions through ApoBD-mediated penetration, we collected ApoBDs from differently treated tumor cells and cocultured them with 4T1-SP spheroids. The results demonstrated that A-(HRA@D-TT) effectively facilitated the ApoBD-mediated neighboring effect, enabling sustained drug delivery to previously inaccessible deep-seated CSCs (fig. S27, E and F). Then, the in vivo validation following the protocol outlined in fig. S27G revealed that, while 4T1-SP tumors exhibited aggressive growth resistant to DOX monotherapy, HRA@D-TT treatment significantly suppressed tumor progression after four cycles (fig. S27, H to J). Furthermore, HRA@D-TT substantially reduced the CD44^+^/CD24^−^ cell population (fig. S27, K and L) and down-regulated CD133 expression, as confirmed by immunofluorescence staining (fig. S27M).

Beyond establishing drug-resistant models through cell sorting, long-term exposure of tumor cells to chemotherapeutic agents has also been shown to induce CSC enrichment. These CSCs can promote therapeutic resistance through various mechanisms, including the up-regulation of STAT3 and nuclear factor erythroid 2–related factor 2 expression (*52*, *53*). Consequently, DOX-resistant 4T1 cells (4T1/DOX), generated through exposure to increasing concentrations of DOX, were also used as a drug-resistant model in this study. As depicted in fig. S28A, parental 4T1 cells and 4T1/DOX cells exhibited distinct cellular morphologies, with the latter transitioning to a mesenchymal-like phenotype. Furthermore, prolonged DOX exposure resulted in a substantial increase in the CSC-like population, correlating with a sixfold increase in DOX resistance (IC_50_ = 578.4 nM) (fig. S28, B and C). Notably, treatment with HRA@D-TT partially restored the cytotoxicity of DOX and reversed drug resistance in 4T1/DOX cells (IC_50_ = 256.8 nM). To investigate the effect of HRA@D-TT on highly drug-resistant cells within the CSC niche, ApoBDs were collected from cells subjected to various treatment conditions and subsequently coincubated with 4T1/DOX tumorsphere cells. As illustrated in fig. S28 (D and E), A-(HRA@D-TT) treatment reduced the proportion of SP cells within the spheroids and inhibited drug efflux. The in vivo therapeutic efficacy of HRA@D-TT was further validated in 4T1/DOX tumor-bearing mice, according to the established protocol (fig. S28F). As depicted in fig. S28 (G to I), DOX monotherapy demonstrated negligible antitumor efficacy against the inherently resistant implanted tumors. Conversely, HRA@D-TT effectively suppressed tumor growth over the 18-day treatment period, maintaining tumor volumes below ~500 mm^3^. Furthermore, treatment with HRA@D-TT resulted in a significant reduction in the population of CD133^+^ CSCs within the tumor (fig. S28J). These results demonstrated that the HRA@D-TT–mediated cascade strategy effectively eliminated CSCs and overcame therapeutic resistance through its deep penetration and differentiation capabilities.

### Boosting antitumor immunity via HRA@D-TT–induced MDSC elimination

**Fig. 8.**
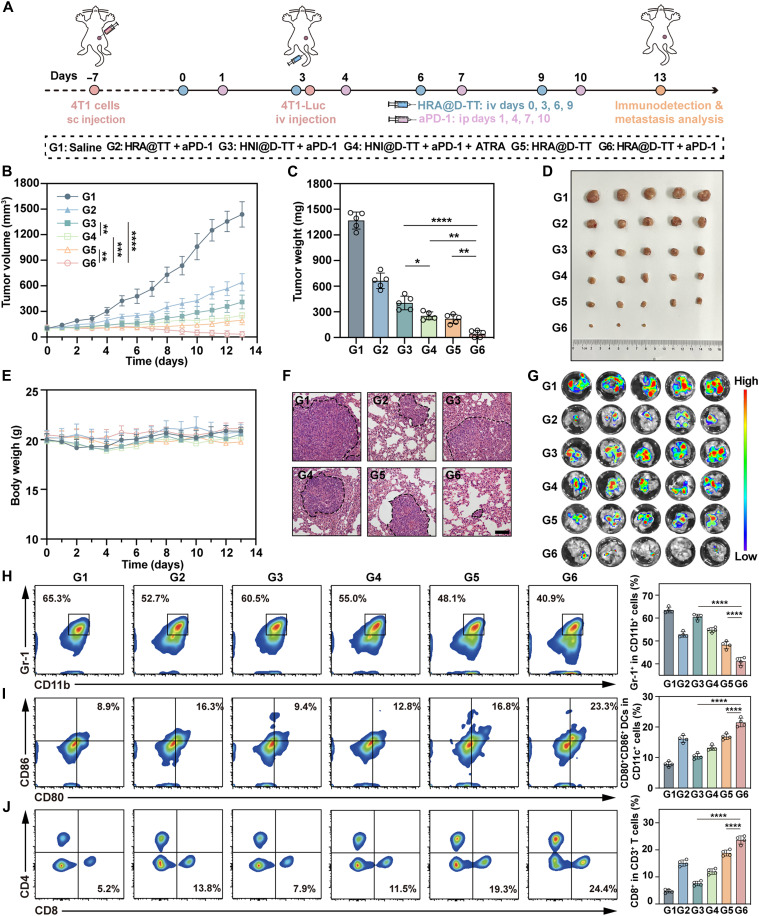
In vivo antitumor immunity enhancement by HRA@D-TT in 4T1 tumor model. (**A**) Schema showing the experiment protocol for immunity response evaluation in 4T1 xenograft model. sc, subcutaneous; iv, intravenous. (**B**) Average tumor growth curves, (**C**) tumor weight, (**D**) tumor photos, and (**E**) body weight of mice after various treatments (*n* = 5 mice). (**F**) H&E-stained images and (**G**) fluorescence images of the lung sections at the end of treatments. (**H**) Flow cytometry measurement of CD11b^+^Gr-1^+^ MDSCs in tumors (*n* = 4 mice). (**I**) Flow cytometry measurement of CD11c^+^CD80^+^CD86^+^ DCs in TDLNs (*n* = 4 mice). (**J**) Flow cytometry measurement of CD3^+^CD8^+^ T cells in tumors (*n* = 4 mice). Data are represented as means ± SD and statistical significance was analyzed through one-way ANOVA. *P < 0.05; **P < 0.01; ***P < 0.001; ****P < 0.0001.

MDSCs reside within tumors and act as immunosuppressive cells, impairing the function of CD8^+^ T cells. Increasing evidence suggested that ATRA could promote the differentiation of MDSCs and enhance the intratumoral infiltration of CD8^+^ T cells (*54*, *55*). To explore this, we investigated the antitumor immune efficacy of HRA@D-TT combined with aPD-1 in a more aggressive metastatic tumor–bearing mice model. The administration protocol was shown in Fig. 8A. On day 10 after establishing the subcutaneous tumor model, 4T1-Luc cells were injected intravenously to simulate the hematogenous metastasis of advanced tumors. Various formulations and aPD-1 antibodies were administered intravenously and intraperitoneally, respectively. As shown in Fig. 8 (B to D), the combination of aPD-1 with HRA@D-TT enhanced antitumor efficacy and even achieved tumor regression. The physical wrap of ATRA increased the tumor inhibition rate of chemoimmunotherapy to 35%, compared to the combination of HNI@D-TT and aPD-1, underscoring ATRA’s potent sensitizing effect. Notably, the antitumor efficacy of coadministering free ATRA with HNI@D-TT and aPD-1 was markedly inferior to that of the HRA@D-TT plus aPD-1 regimen, highlighting the crucial role of the sequential drug release platform in ensuring optimal ATRA therapeutic activity. Additionally, the treatment maintained the mice’s weight within a stable range, indicating the biosafety of the HRA@D-TT and aPD-1 combination (Fig. 8E). Subsequently, the lungs were collected to assess the extent of metastasis through H&E staining and bioluminescence. As shown in Fig. 8 (F and G) and fig. S29, metastasis to the lungs was significantly inhibited after treatment with HRA@D-TT + aPD-1, indicating that the combination treatment significantly curtailed metastatic tumor progression.

Subsequently, the number of infiltrating immune cells in the collected tumors and tumor-draining lymph nodes (TDLNs) was quantified using flow cytometry. MDSCs comprised more than 60% of the CD11b^+^ cells in untreated 4T1 tumors, a finding consistent with the “cold” tumor characteristics of 4T1 (*56*). Treatment with HRA@D-TT + aPD-1 reduced the MDSC proportion by half, whereas HNI@D-TT + aPD-1 exhibited a less marked effect (Fig. 8H). Furthermore, MDSCs can be categorized into two subsets, monocytic MDSCs (M-MDSCs) and polymorphonuclear MDSCs (PMN-MDSCs), based on morphological and functional distinctions. M-MDSCs are known to primarily exert immunosuppressive functions within the TME through the production of immunosuppressive factors such as TGF-β. As depicted in fig. S30 (A, F, and G), HRA@D-TT primarily targeted M-MDSC differentiation, leading to an approximately sixfold reduction in their proportion within tumor tissues. Concurrently, a diminished presence of PMN-MDSCs in tumor tissues was also observed, potentially attributable to a synergistic effect of DOX-mediated cytotoxicity and ATRA-induced differentiation. Further flow cytometric analysis revealed that HRA@D-TT + aPD-1 administration enhanced dendritic cell (DC) maturation and increased the frequency of proinflammatory M1 macrophages (Fig. 8I and fig. S30, B, C, H, and I). In addition, a significant infiltration of CD8^+^ T cells, crucial for antitumor immunity, was observed following treatment with HRA@D-TT and HRA@D-TT + aPD-1 (Fig. 8J). Consistent with the increased CD8^+^ T cell frequency, an elevated proportion of activated cytotoxic T lymphocytes (CTLs) (IFN-γ^+^CD8^+^ T cells and CD69^+^CD8^+^ T cells) was detected in tumors, significantly exceeding that observed in the HNI@D-TT + aPD-1 and HNI@D-TT + aPD-1 + ATRA treatment groups (fig. S30, D, E, J, and K). These findings underscored the critical role of the differentiating agent ATRA and the hypoxic core in the rational design of sequential drug delivery platforms. In contrast, HNI@D-TT + aPD-1 and HNI@D-TT + aPD-1 + ATRA demonstrated limited efficacy in overcoming the MDSC-mediated immune barrier, thereby restricting the immune response of aPD-1 in solid tumors.

In addition, cytokine secretion in tumors was analyzed through enzyme-linked immunosorbent assay (fig. S31). Tumors treated with HRA@D-TT exhibited increased secretion of tumor necrosis factor–α (TNF-α) and interferon-γ (IFN-γ), while the TGF-β and IL-10 levels were reduced. These results indicated the combined treatment successfully reversed immunosuppression and reactivated the antitumor activity of aPD-1. Notably, the HRA@D-TT application increased the level of CD8^+^ T cells in the blood, which indicated that the combination of the nanoplatform and aPD-1 elicited a strong systemic antitumor immune response (fig. S32). Overall, these findings suggested that the ATRA-encapsulating nanoplatform can improve immune cell infiltration by differentiating MDSCs. In particular, in conjunction with aPD-1, it demonstrated superior immune activation and tumor suppression capabilities.

### HRA@D-TT + aPD-1 remodels the T cell immune within CT26 tumor models

**Fig. 9.**
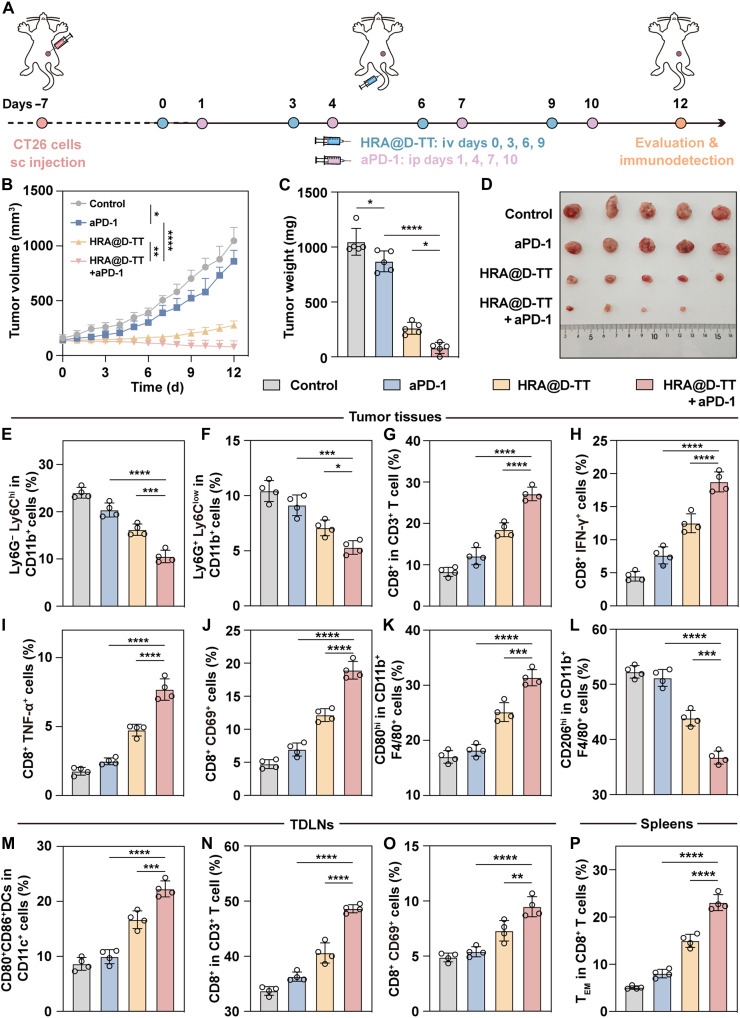
HRA@D-TT + aPD-1 reverses immunosuppression and activates T cells in CT26 tumor models. (**A**) Schedule of the HRA@D-TT combined with aPD-1 experiment in CT26 tumor-bearing mice. (**B**) Tumor volume curves of different treatments (*n* = 5 mice). d, days. (**C**) Tumor weight of the mice after the final treatment (*n* = 5 mice). (**D**) Images of tumors after 12 days of treatment (*n* = 5 mice). Quantitative analysis of (**E**) M-MDSCs, (**F**) PMN-MDSCs, (**G**) CD8^+^ T cells, (**H**) IFN-γ^+^CD8^+^ CTLs, (**I**) TNF-α^+^CD8^+^ CTLs, (**J**) CD69^+^CD8^+^ CTLs, (**K**) M1 macrophages, and (**L**) M2 macrophages in tumor tissues (*n* = 4 mice). Quantitative analysis of the infiltration of (**M**) DCs, (**N**) CD8^+^ T cells, and (**O**) CD69^+^CD8^+^ CTLs in TDLNs (*n* = 4 mice). (**P**) Quantitative of the proportion of T_EM_ in spleens (*n* = 4 mice). Data are represented as means ± SD and statistical significance was analyzed through one-way ANOVA. *P < 0.05; **P < 0.01; ***P < 0.001; ****P < 0.0001.

To investigate the applicability of chemo-immunotherapy combinations in diverse tumor types, we established a CT26 tumor model according to the protocol outlined in Fig. 9A. As illustrated in Fig. 9 (B to D), the combination therapy demonstrated significant antitumor efficacy, resulting in tumor regression. In contrast, while HRA@D-TT substantially inhibited tumor progression, aPD-1 monotherapy merely decelerated growth. To evaluate the mobilization of antitumor immunity by different treatments in vivo, we harvested tumors and TDLNs on day 12. Immune cell infiltration and proportional changes were subsequently analyzed via flow cytometry. Our results revealed that HRA@D-TT reduced the proportions of both M-MDSCs and PMN-MDSCs, with a particularly marked reduction in highly immunosuppressive M-MDSCs. In comparison, aPD-1 monotherapy exhibited less pronounced effects (Fig. 9, E and F, and fig. S33A). Furthermore, combination therapy induced substantial infiltration of CD8^+^ T cells and high proportions of activated CTLs, as evidenced by increased frequency of CD69^+^CD8^+^, IFN-γ^+^CD8^+^, and TNF-α^+^CD8^+^ T cells (Fig. 9, G to J, and fig. S33, B to E). These findings suggested that the combination therapy maximized the T cell activation capacity of aPD-1 by concurrently inhibiting MDSCs, thereby remodeling the immunosuppressed TME. Moreover, as shown in Fig. 9 (K and L) and fig. S33 (F and G), HRA@D-TT alone and in combination with aPD-1 promoted MDSC differentiation, facilitating the repolarization of tumor-associated macrophages toward proinflammatory M1 phenotypes. The attenuated immunosuppressive tumor properties also induced the reorganization of immune cells within TDLNs, characterized by enhanced DC maturation and elevated CD8^+^ T cell activation (Fig. 9, M to O, and fig. S33, H to J). Additionally, as shown in Fig. 9P and fig. S33K, treatment altered splenic memory T cell composition, shifting the balance from central memory T cells (T_CM_) toward effector memory T cells (T_EM_). These dynamic immunological modifications indicated that the HRA@D-TT–mediated chemo-immunotherapy combination facilitated rapid T cell immunity initiation, successfully activating antitumor immune responses across both local and systemic compartments. Collectively, our findings demonstrated that HRA@D-TT, either as monotherapy or in combination with ICIs, represented a promising therapeutic strategy that effectively reversed immunosuppressive microenvironments, enhanced T cell functionality, and promoted durable antitumor immunity across multiple cancer models.

### Comparative study of the antitumor efficacy of different nano-formulations

To elucidate the distinct advantages of HRA@D-TT, we investigated the therapeutic effects of various nano-sized formulations. Commercially available DOX liposomes (Doxil) and laboratory-prepared poly(ethylene glycol)-block-poly(lactic acid) (PEG-b-PLA) nanoparticles (DA NPs) were selected as reference formulations because of their established validation and widespread use (fig. S34A). We assessed their antitumor efficacy in 4T1-bearing mice following the protocol outlined in fig. S34B. As depicted in fig. S34 (C to E), HNI@D-TT exhibited an effect comparable to Doxil in monotherapy regimens. Upon treatment completion, HNI@D-TT and Doxil reduced tumor volumes approaching 400 mm^3^, compared to ~1000 mm^3^ in the saline group. Furthermore, HRA@D-TT demonstrated a significant tumor suppression advantage over DA NPs and substantially prolonged survival in a two-agent synergistic treatment. Flow cytometric analysis of tumor tissues revealed that HRA@D-TT had substantial advantages in reducing immunosuppressive M-MDSC/PMN-MDSC populations and targeting CSCs (fig. S34, F to I). This suggested that HRA@D-TT effectively leveraged the differentiation-inducing effects of ATRA, thereby dismantling immunosuppressive and chemo-resistant barriers within solid tumors.

Moreover, we substituted ATRA with DiR in the formulation and observed tumor sections via CLSM. As shown in fig. S34J, most of HDiR@D-TT–delivered DiR predominantly localized to the hypoxic region (regions enriched with green fluorescence), with a minor portion diffusing into the normoxic region (regions deficient in green fluorescence). This demonstrated the enhanced accessibility of internally encapsulated drugs to cells within the hypoxic zones. Conversely, DiR encapsulated in PLA nanoparticles exhibited limited penetration beyond the normoxic region, failing to overcome the hypoxic barrier. These findings substantiated that HRA@D-TT facilitated spatially controlled drug release specifically within hypoxic microenvironments, thereby significantly enhancing the therapeutic efficacy of differentiation therapy by targeting resistant tumor regions typically inaccessible to conventional drug delivery systems.

Although HNI@D-TT and Doxil demonstrated similar efficacy, their in vivo behavior differed substantially. As illustrated in fig. S34K, HNI@D-TT enhanced the infiltration of the chemotherapeutic agent into the tumor, resulting in a more homogenous distribution of DOX fluorescence. Conversely, Doxil fluorescence remained confined to perivascular tumor areas without reaching avascular regions. The advantageous properties of Doxil may stem from its extended circulation time in vivo, leading to greater drug accumulation at the tumor site via the EPR effect. Nevertheless, it is crucial to recognize that this PEG-conferred prolonged circulation also contributes significantly to Doxil’s dermatological toxicity manifestations, such as hand-foot syndrome (*57*).

## DISCUSSION

The significant infiltration of CSCs and MDSCs into the deep hypoxic areas of tumors is a key factor contributing to chemotherapy resistance and immune escape, resulting in unsatisfactory chemoimmunotherapy outcomes. ATRA presents a promising approach by promoting differentiation of CSCs toward mature phenotypes with reduced stemness characteristics, while simultaneously inducing MDSCs to differentiate into immunologically active DCs and macrophages. Nevertheless, effectively delivering differentiation agents to deep tumor niches remains unresolved, as the dense extracellular matrix and elevated interstitial fluid pressure constrain drug penetration.

ApoBDs function as reservoirs for residual intracellular therapeutics following cellular apoptosis, mediating successive intercellular drug delivery through the neighboring effect, thereby inducing broader cytotoxic effects. Nevertheless, this process is primarily limited by the proportion of unbound active compounds available for subsequent penetration cycles. Our investigations revealed a progressive decrease in unbound active drug concentration during extended penetration. Furthermore, unregulated burst release resulted in excessive drug consumption within individual cells, compromising sustained apoptotic induction during subsequent penetration phases. By designing a pulsatile drug release platform to regulate the drug release kinetics, we achieved active drug retention and maintained prolonged tumor toxicity in deeper regions through ApoBD-mediated delivery.

For effective differentiation and elimination of tumor-embedded CSCs, we demonstrated that deeply situated CSCs efficiently internalized transported ApoBDs, facilitating the intracellular accumulation of therapeutic agents contained within these vesicular structures. This characteristic significantly enhanced nanomedicine accessibility to typically resistant CSC populations, augmenting chemotherapeutic cytotoxicity and differentiation agent efficacy. Moreover, this approach validated the feasibility of leveraging in situ–generated apoptotic vesicles for comprehensive CSC eradication. Our strategy additionally addressed tumor spatial heterogeneity through sequential drug release mechanisms targeting distinct tumor regions. The approach involved initial DOX release to trigger ApoBD-mediated penetration while preventing premature ATRA leakage in peripheral tumor zones. Upon reaching deeper hypoxic regions, the platform enabled specific ATRA release targeting infiltrated CSCs and MDSCs, thereby simultaneously overcoming chemoresistance and reinvigorating antitumor immunity.

In summary, this research developed a pulsatile sequential drug release nanoplatform that integrated active tumor penetration and differentiation therapy to simultaneously boost chemotherapy and immunotherapy. This nanoplatform exhibited responsive behavior to both intracellular stimuli and hypoxic microenvironments, enabling precisely controlled therapeutic agent release that effectively addressed cellular spatial heterogeneity within tumors. Our findings demonstrated that lysosomal-to-cytoplasmic pH variations triggered controlled DOX release, enhancing ApoBD-mediated penetration into deeper tumor regions. The subsequent hypoxia-responsive release of ATRA achieved dual depletion of CSCs and MDSCs, synergistically countering chemoresistance and immune suppression mechanisms. Collectively, the cascade strategy of ApoBD-inspired penetration and targeted differentiation therapy demonstrated significant potential in various tumor models, representing an optimal therapeutic approach to enhance the efficacy of current chemo-immunotherapy regimens.

## MATERIALS AND METHODS

### Materials

DOX, ATRA, 2-nitroimidazole, *N*-hydroxysuccinimide (NHS), and 1-ethyl-3-(3-dimethyl aminopropyl)carbodiimide hydrochloride (EDCI) were purchased from Aladdin Bio-Chem Technology Co. Ltd. (Shanghai, China). TA and TEPA were purchased from Macklin Reagent Co. Ltd. (Shanghai, China). HA (90 kDa), DiR, Hoechst 33342, MTT, fetal bovine serum (FBS), cell culture medium, and the reagents used in Western blotting were obtained from Meilun Biotech Co. Ltd. (Dalian, China). Doxorubicin liposome (Doxil) was obtained from Cspc Ouyi Pharmaceutical Co. Ltd. (Shijiazhuang, China). The following antibodies were supplied by BioLegend, including anti–PD-1, phycoerythrin (PE)–conjugated anti-CD44, fluorescein isothiocyanate (FITC)–conjugated anti-CD24, allophycocyanin–cyanine 7 (APC-Cy7)–conjugated anti-CD45, BV421-conjugated anti–Gr-1, PE-conjugated anti–Ly-6C, BV421-conjugated anti–Ly-6G, APC-conjugated anti–TNF-α, PE-conjugated anti–IFN-γ, BV421-conjugated anti-CD69, FITC-conjugated anti-CD11b, FITC-conjugated anti-CD11c, PerCP-Cy5.5–conjugated anti-F4/80, APC-conjugated anti-CD206, PE-conjugated anti-CD80, APC-conjugated anti-CD86, FITC-conjugated anti-CD3, APC-conjugated anti-CD4, PerCP-Cy5.5–conjugated anti-CD8, and FITC-conjugated anti-CD133.

### Synthesis of NI-NH

2-Nitroimidazole (304 mg, 2.7 mmol) was dissolved in dimethyl formamide (DMF; 12 ml), and potassium carbonate (K_2_CO_3_, 555 mg, 4 mmol) was added to the reaction mixture. The mixture was stirred for 1 hour at 80°C, and a solution of 6-(Boc-amino) hexyl bromide (790 mg, 2.8 mmol) in 2 ml of DMF was then added dropwise. After stirring for an additional 4 hours, the mixture was filtered to remove insoluble solids and washed with ethanol. The crude product was obtained by rotary evaporation of the residual solution and further purified by extraction with ethyl acetate and water. The Boc-protected 6-(2-nitroimidazole) hexylamine (NI-NH) was obtained by evaporating the organic layer (compound a). Subsequently, the Boc-protected NI-NH was redissolved in methanol on ice and stirred for 24 hours in a 1.25 M hydrogen chloride-methanol (CH_3_OH-HCl) solution to remove the Boc group, yielding NI-NH (compound b).

### Synthesis of HA-grafted nitroimidazole (HNI) polymer

HA (200 mg) was dissolved in water, and EDCI (537 mg, 2.8 mmol) and NHS (345 mg, 3 mmol) were added to activate the carboxyl groups under ice bath conditions for 30 min. To this solution, NI-NH (149 mg, 0.28 mmol) in 4 ml of DMF was added dropwise with continuous stirring. After a 24-hour reaction, the reaction solution was first dialyzed against a 1:1 water/methanol mixture for 24 hours, followed by an additional 48 hours of dialysis against water. Subsequently, the nitroimidazole-grafted HA (HNI, compound c) was obtained through lyophilization.

### Preparation and characterization of HRA@D-TT

The preparation of HRA@D-TT was carried out in two steps. First, to fabricate the ATRA-loaded nanocore (HRA), ATRA (2 mg) was dissolved in 1 ml of chloroform, and HNI (13 mg) was dissolved in 5 ml of PBS. The organic solvent was dropwise into the aqueous solution and sonicated for 20 min. The organic solvent was eliminated using a rotary evaporator, and the nonencapsulated drugs were removed via filtration using a porous membrane with a pore size of 220 nm. For quantifying the internal core-loaded ATRA, the nanoparticle solution was diluted with methanol and sonicated in an ice bath for 20 min. Following centrifugation, the ATRA content in the supernatant was determined by high-performance liquid chromatography (HPLC). In addition, HNI micelles without drugs encapsulated were obtained by directly dissolving the polymer in PBS with ultrasonication.

To form the TA-TEPA shell, the freeze-dried HRA (20 mg) were redispersed in tris-HCl buffer (10 ml, pH 8.5), followed by the addition of TEPA (2 μl) and TA (5 mg), and stirred overnight under dark conditions. The solid product (HRA@TT) was then centrifuged, washed three times, and freeze-dried. For DOX loading, triethylamine was first added to the DMF with DOX·HCl dissolved at a molar rate of 3:1 (triethylamine:DOX). After vertexing for 1 hour, the mixture was evaporated under vacuum and then redissolved in dimethyl sulfoxide (DMSO). Next, HRA@TT (100 mg) was dissolved in deionized water, and the DOX mixture was added dropwise, followed by incubation for 2 hours. HRA@D-TT was obtained via centrifugation and washing three times. To measure the DL efficiency of DOX, the supernatant was collected after centrifugation, and its fluorescence intensity was analyzed at an excitation wavelength of 480 nm using a Varioskan Flash multimode microreader (Thermo Fisher Scientific, USA). The concentration of DOX in the supernatant was determined according to a standard calibration curve. The DL of DOX was then calculated using the following formula: drug loading (%) = $\frac{m_{\text{DOX}}-c_{1}v_{1}}{m_{0}}\times 100\%$ , *m*_DOX_ is the initial mass of DOX added, *c*_1_ is the concentration of DOX in the supernatant, *v*_1_ is the volume of the supernatant after centrifugation, and *m*_0_ is the mass of the dried nanoplatform. Pure TA shells were obtained by dispersing HNI or HRA nanoparticles in a tris-HCl (pH 7.4) solution containing TA (0.5 mg/ml).

TEM (Hitachi, HT7700, Japan) was used to visualize the morphology of HRA@D-TT and HRA. The hydrodynamic diameter and zeta potential of the HRA@D-TT and HRA@D-TA at different pH values were measured with Zetasizer (Nano ZS, Malvern Co., UK). Each sample was prepared to 1 mg/ml in PBS (pH 7.4 or 5.0) and incubated for 6 hours before measurement. FTIR (Tensor 27, Bruker Co. Ltd., Germany), XPS (Escalab 250Xi, Thermo Fisher Scientific, UK), and fluorescence spectroscopy (RF-5301PC, SHIMADZU, Japan) were used to confirm the shell binding and DOX encapsulation. The fluorescence spectra of DOX and HNI@D-TT (DOX, 2 μg/ml) were measured across the 500- to 700-nm range with an excitation wavelength of 470 nm. The colloidal stability was investigated by measuring particle size after incubation of HRA@D-TT and HRA@D-TA (1 mg/ml) in PBS with 10% FBS supplemented for 48 hours at 37°C. Additionally, the long-term stability was evaluated by storing the nanoparticles at 4°C for 2 weeks.

The bicinchoninic acid (BCA) assay was used to quantify the TA content on the surface of the nanoparticles. HRA@D-TT and HRA@D-TA were first incubated in PBS at pH 7.4 for 6 hours, followed by incubation in PBS at pH 5.0 for another 6 hours, constituting one incubation cycle. The supernatant from each incubation cycle was mixed with the BCA reagent, followed by the absorption at 562 nm was measured. The remaining TA on the nanoparticle surface was calculated using the following equation: $\text{The remaining}\;\text{TA}\;\left(\%\right)=\frac{m_{\text{TA}}-△m}{m_{\text{TA}}}\times 100\%$ , where △*m* is the mass of TA in the supernatant after each round of treatment and *m*_TA_ is the initial mass of TA on the nanoparticle surface, determined after 2-hour incubation with the BCA reagent.

### In vitro FRET analysis

For the FRET nanoplatform preparation, DiI and DiO were coencapsulated in the HNI polymer at a feeding ratio of 1:1:10 (DiI:DiO:HNI, w/w/w) via the emulsion solvent evaporation method. Under the normoxic or simulated hypoxic environment, 4T1 cells were cultured in 24-well plates (2 × 10^4^ cells per well) overnight and then further treated with HNI-(DiI + DiO)@TT for 8 hours at a dye concentration of 2 μg/ml each. Excess micelles were removed by washing the cells three times with cold PBS, followed by 4% formaldehyde fixing and Hoechst 33342 staining. CLSM (Nikon C2 plus) images were acquired with an excitation wavelength of 488 nm, using 505- to 550-nm filters for green emission and 560- to 615-nm filters for red (FRET) emission. Furthermore, the cellular fluorescence was quantitatively assessed via ImageJ.

### In vitro drug release

The in vitro release profile of HRA@D-TT and HRA@D-TA was investigated via dialysis. In brief, HRA@D-TT and HRA@D-TA (1 ml) were placed into a dialysis bag [molecular weight cut-off (MWCO), 3.5 kDa] and immersed in 30 ml of PBS containing 2% Tween 80. To assess the pH-induced DOX release, the medium was adjusted into a series of acidity gradients (pH 7.4, 6.8, and 5.0). In addition, an oxygen-deprived system was also established to evaluate the hypoxia-responsive release of ATRA from HRA@D-TT by adding sodium dithionite (Na_2_S_2_O_4_, 10 mM) into the acid buffers. Samples of 200 μl were withdrawn at predetermined intervals and immediately replaced with fresh medium of the same volume. The released DOX and ATRA from nanoplatform were monitored by fluorescence spectrometer and HPLC, respectively.

The conversion of nitroimidazole to aminoimidazole group was validated under the hypoxic conditions. HNI was dispersed into PBS with or without Na_2_S_2_O_4_ and incubated for 8 hours under shaking. The absorbance spectra of HNI were then recorded with a microplate reader.

### pH-dependent drug release under alternating conditions

HRA@D-TT and HRA@D-TA (1 ml) were loaded into a dialysis bag and subsequently suspended in 30 ml of PBS (pH 7.4, containing 2% Tween 80). The system was maintained at 37°C for 6 hours. Subsequently, the pH of the release medium was adjusted to 5.0, and the incubation was continued for an additional 6 hours. At predetermined time points, 200 μl of samples were collected from the release medium, and the concentration of DOX was quantified using fluorescence spectrometry. This pH-alternating incubation process was defined as one release cycle. The cycle was repeated until the amount of DOX released per cycle decreased to less than 5%.

### Cell culture

4T1 cells were sourced from the Chinese Academy of Sciences (Beijing, China). Luciferase-tagged 4T1 cells (4T1-Luc) were supplied by Shanghai Zhongqiao Xinzhou Biotechnology Co. Ltd. (Shanghai, China). Both cell lines were cultured in RPMI-1640 supplemented with 10% FBS, streptomycin sulfate (100 μg/ml), and penicillin (100 U/ml). The cells were maintained in an incubator at 37°C with 5% CO_2_.

### Cellular uptake and lysosome escape

4T1 cells were seeded in 24-well plates (2 × 10^4^ cells per well) and cultured for 12 hours to allow for attachment. Subsequently, the cells were treated with fresh medium containing Free DOX, HNI@D-TT, and HNI@D-TA (5 μg/ml, DOX equivalent) for 4 and 8 hours, respectively. After treatment, the cells were fixed using 4% paraformaldehyde, and the nuclei were stained with Hoechst 333342. Following three washes, CLSM was applied to detect the cellular uptake, and flow cytometry (BD FACSCelesta) provided quantitative analysis.

The lysosome escape behaviors were assessed similarly. The attached cells were incubated with HNI@D-TT or HNI@D-TA containing DOX at 5 μg/ml for 4 hours and further counterstained with LysoTracker green and Hoechst 33342. After washing off the dye, CLSM was used for observation.

### Cytotoxicity assay

To evaluate the in vitro cytotoxicity against tumor cells, 4T1 cells were plated in 96-well plates at a density of 2000 cells per well. After 12 hours, the medium was replaced with gradient dilutions of DOX, HNI@D-TT, HNI@D-TA, and HRA@D-TT. After incubating in normoxic or hypoxic atmospheres for 48 hours, the MTT solution was added and incubated for 4 hours at 37°C. To dissolve the formazan crystals, 200 μl of DMSO was added to each well, and the absorption at 570 nm was detected using a microplate reader. The IC_50_ values were determined using GraphPad Prism 8 on the basis of molar concentration and cell viability ratio.

### Isolation and characterization of ApoBDs

ApoBDs were isolated via sequential centrifugation as previously reported. First, 4T1 cells were cultured in six-well plates and treated with HNI@D-TT (5 μg/ml, DOX equivalent) for 8 hours. After a 12-hour incubation with fresh medium, the culture was harvested and subjected to preliminary clarification by low-speed centrifugation (300*g* for 10 min at 4°C using a fixed-angle rotor) to pellet cellular debris and intact cells. The resulting supernatant was carefully aspirated and transferred to fresh polypropylene tubes, followed by a second centrifugation at 3000*g* for 20 min at 4°C to concentrate larger membrane-bound particles. The ApoBDs pellet was gently resuspended in 1 ml of ice-cold PBS supplemented with protease inhibitor. To investigate the cellular uptake of ApoBDs, 4T1 cells were incubated with ApoBDs for 8 hours. After staining the nuclei, cells were observed with CLSM. TEM was used to observe the morphology of ApoBDs, and a Western blot experiment was performed for characteristic protein verification. Annexin V^+^ ratios for ApoBDs were determined using an Annexin V-FITC/PI kit (Solarbio) and analyzed by flow cytometry.

### Intercellular delivery of HRA@D-TT

4T1 cells were seeded in the six-well plates (i-iii) and incubated overnight. The cells in well (i) were treated with Free DOX, HNI@D-TT, and HNI@D-TA (5 μg/ml, DOX equivalent) for 8 hours under normoxia (the cell state was defined as i-I), followed by a 12-hour incubation in fresh medium to induce apoptosis (the cell state was defined as i-A). Then, ApoBDs were collected from the supernatant and coincubated with untreated fresh cells in well (ii) for 8 hours. This process was repeated three times to obtain treated cells in the well (iii). DOX internalization was assessed by observing with CLSM after washing the cells and staining with Hoechst 33342. To evaluate apoptosis, the treated cells were subjected to stain with an annexin V-FITC/PI kit, and the cell apoptosis rate was examined by flow cytometry.

Additionally, we quantified the free drug ratio in cells and ApoBDs, which were collected for ice bath ultrasonication and protein precipitation with methanol. After centrifugation and ultrafiltration, the DOX content in the filtrate was measured using a microplate reader and normalized to their protein content.

To analyze the behavior of the drug encapsulated in hypoxic cores during the intercellular transfer process, coumarin 6 was encapsulated into the inner core of HNI@D-TT and incubated with cells in well (i). After repeating three times, ApoBDs (i-ii) and (ii-iii) were extracted from cell culture and visualized by CLSM after dropping onto slides.

### Penetration in 3D tumor spheroids

The tumor tissue-mimicked 3D spheroids were established through the hanging drop method. Briefly, 4T1 cell suspension was mixed with 0.24% methylcellulose (M0512, Sigma-Aldrich) at a ratio of 1:1, and 15 μl of the mixture was pipetted onto the lid of a round-bottom 96-well plate. The lid with cell suspension droplets was inverted over the plate containing culture medium and incubated at conventional culture conditions for 24 hours and then centrifuged at 2800 rpm for 3 min. After 6 days of culture, tumor spheroids were exposed to Free DOX, HNI@D-TT, and HNI@D-TA (5 μg/ml, DOX equivalent) for 24 hours. Z-stack imaging at 40 μm intervals was used to characterize DOX fluorescence in spheroids via CLSM.

### Enrichment and culture of BCSCs

As previously described, 4T1 cells were seeded in ultralow attachment six-well plates (2 × 10^4^ cells per well, Corning) and cultured in serum-free DMEM/F12 medium supplemented with epidermal growth factor (20 ng/ml), basic fibroblast growth factor (20 ng/ml), and B27 solution (1×). Passaging is performed every 7 days, and the third-generation suspended spheres are collected via low-speed centrifugation to obtain a highly enriched population of CSCs.

### In vitro CSC uptake of ApoBDs

4T1 cells in ultralow attachment six-well plates (2 × 10^4^ cells per well) were cultured to form tumorspheres. Free DOX, HNI@D-TT, and ApoBDs (from HNI@D-TT–induced apoptosis) were added to the culture medium at the equivalent DOX concentration of 5 μg/ml. After incubation for 4 hours, tumorsphere cells were counterstained with Hoechst 33342 for CLSM detection, with fluorescence intensity quantified by flow cytometry. To investigate the underlying mechanisms, AFM was used to assess the surface roughness of the cells fixed with 2.5% glutaraldehyde after 4-hour treatment. We also analyzed P-gp expression by the Western blot experiment.

### Anti-CSC assay

Tumorspheres from the third-generation cultures were digested into single cells and re-inoculated into ultralow attachment six-well plates (2 × 10^4^ cells per well). After overnight incubation, the cells were treated with various ApoBDs under hypoxia for 48 hours and then centrifuged to gain cell samples. To identify CSCs, cells were stained with CD44-PE and CD24-FITC for 40 min at 4°C. For SP analysis, 4T1 cells were seeded in six-well plates and treated with ApoBDs for 48 hours in hypoxia. Afterward, cells were incubated with Hoechst 33342 (5 μg/ml) for 1 hour at 37°C followed by PBS washing. Verapamil hydrochloride (100 μM) was used as a control. The proportions of CD44^+^/CD24^−^ cells in the tumorspheres and SP cells in 4T1 cells were determined using a flow cytometer.

For the tumorsphere formation assay, 4T1 cells were pretreated with different ApoBDs for 48 hours in hypoxia and then recultured in ultralow attachment 24-well plates (Corning) at a density of 2 × 10^4^ cells per well following the established approach. After a 7-day culture period, the spheres from each treatment were imaged and counted.

### Western blot analysis

Western blot experiment was performed to assess the expression of stem-related proteins. For the comparison of Sox2, Nanog, Oct4, and P-gp expression among various treatments, cell samples were lysed after treatment. Samples with equal amounts of protein were electrophoretically separated via SDS-PAGE followed by transferred on polyvinylidene difluoride membranes (Bio-Rad). Afterward, the membranes were blocked with tris-buffered saline with Tween 20 (TBST) buffer containing 5% skim milk for 2 hours. Then, the films were incubated overnight with primary antibodies, including Sox2 (Abcam, ab92494), Nanog (Abcam, ab109250), Oct4 (Abcam, ab181557), and P-gp (Abcam, ab170904). Following incubation, the membranes were washed with TBST and further treated with horseradish peroxidase–linked secondary antibody for 1 hour. Protein bands were detected using chemiluminescent (ECL) detection reagents.

### Animal studies

Female BALB/c mice and female C57BL/6 mice (aged 6 to 8 weeks) were supplied by the Laboratory Animal Center of Shenyang Pharmaceutical University (Shenyang, China). All experimental procedures involving animals were conducted in compliance with the Care and Use of Laboratory Animals guidelines of Shenyang Pharmaceutical University and received approval from the University’s Animal Ethics Committee (SYPU-IACUC-2024-0311-041).

### In vivo distribution and tumor penetration

To investigate the biodistribution of HRA@D-TT, a 4T1 tumor model was established by subcutaneously injecting with 1 × 10^6^ 4T1 cells. Upon reaching a tumor volume of ~400 mm^3^, mice were allocated into three groups (*n* = 3) and received intravenous injections of free DiR, HDiR@D-TA, and HDiR@D-TT (1 mg/kg equivalent to DiR). At predetermined time points, the biodistribution of DiR-labeled nanoplatform was imaged using an in vivo imaging system (IVIS, PerkinElmer). Twenty-four hours after injection, mice were euthanized, and the major organs (heart, liver, spleen, lung, and kidney) along with tumors were collected for in vivo imaging system (IVIS) imaging. To investigate the nanocore-mediated intratumoral hypoxic targeting ability, the tumor tissues were collected, sectioned, and stained with 4′,6-diamidino-2-phenylindole (DAPI) and HIF-1α for CLSM observation.

Tumor penetration was evaluated by administering free DOX, HNI@D-TA, and HNI@D-TT (2 mg/kg equivalent to DOX) to tumor-bearing mice. After 24 hours, the mice were euthanized, and tumor tissues were collected for section. Then, the sections were subjected to DAPI and CD31 staining for CLSM observation.

### In vitro antitumor efficacy

The 4T1 tumor-bearing mice were constructed by subcutaneously injecting 100 μl of PBS containing 1 × 10^6^ 4T1 tumor cells. When the tumor grew to ~100 mm^3^, the mice were randomized into six groups (G1, saline; G2, DOX; G3, HNI@D-TA; G4, HNI@D-TT; G5, HNI@D-TT + ATRA; and G6, HRA@D-TT). During the treatment, the mice received intravenous injections on days 0, 3, 6, and 9, with each dose containing DOX at an equivalent concentration of 2 mg/kg. Mice were monitored daily for body weight and tumor growth. Tumor volume was determined by measuring the length (*L*) and width (*W*) of subcutaneous tumors with digital calipers and calculated using the standard formula: Volume = (*L* × *W*^2^)/2. After treatment, the major organs along with tumor tissues were dissected for H&E staining. In addition, the apoptotic and proliferative levels of tumor cells were assessed by TUNEL and Ki67 immunofluorescence staining. Blood samples were also collected for hepatorenal function analysis. For flow cytometer analysis of the CSC population, the tumor samples were minced into small pieces and digested in RPMI-1640 medium containing collagenase IV, hyaluronidase, and deoxyribonuclease I. Fluorescence antibodies, CD44-PE and CD24-FITC, were incubated with the resulting cells for 40 min at 4°C. Furthermore, tumor sections were also stained for Sox2, Oct4, and Nanog and observed using CLSM to detect the intratumoral stem-related pluripotency factors expression.

A subcutaneous CT26 tumor model was established by injecting 100 μl of PBS-suspended CT26 cells (1 × 10^6^ cells) into BALB/c mice. Once tumors reached ~100 mm^3^, mice were intravenously administered therapeutic agents (groups G1 to G6) every 3 days for a total of four doses. Tumor volume was recorded daily. On day 12, the mice were euthanized, and tumor tissue was dissected, photographed, weighed, and processed for immunofluorescence staining.

For the orthotopic pancreatic tumor model, 1 × 10^6^ Panc02-Luc cells were surgically implanted into the pancreas of C57BL/6 mice. Ten days postinoculation, tumor-bearing mice (*n* = 5 per group for imaging and *n* = 5 per group for survival analysis) received intravenous injections of the various formulations. Bioluminescence imaging was performed at predetermined time points using an IVIS. Mice designated for imaging (*n* = 5) were euthanized on day 14, and tumors were harvested for CD133 staining. A separate cohort of mice (*n* = 5) was monitored for survival analysis.

### Investigation of HRA@D-TT therapeutic efficacy in drug-resistant models

For the SP-resistant cell model, SP cells were isolated from 4T1 cells via flow cytometric sorting (BD Aria SORP), followed by maintenance in the CSC medium for 7 days. Photomicrographs were obtained to document spheroid morphology. The CSC proportion was quantified through flow cytometric analysis using CD44-PE and CD24-FITC staining. To establish the 4T1/DOX-resistant model, 4T1 cells were subjected to gradually increasing concentrations of DOX (20 to 500 nM) over an extended period. The resultant cells were analyzed via flow cytometry following CD44-PE and CD24-FITC staining to determine the proportion of CSC populations.

For in vivo studies, 1 × 10^6^ 4T1-SP or 4T1/DOX cells were subcutaneously inoculated into BALB/c mice to establish ectopic tumor models. When tumor volumes reached 100 to 200 mm^3^, mice were randomly assigned to six treatment groups: (i) saline, (ii) DOX, (iii) HNI@D-TA, (iv) HNI@D-TT, (v) HNI@D-TT + ATRA, and (vi) HRA@D-TT. Treatments were administered four times in total. Tumor volumes were monitored daily. At the designated endpoint, mice were humanely euthanized, and tumor tissues were harvested for photography, flow cytometric analysis, and immunofluorescence staining.

### In vivo antitumor immune responses

4T1 tumor-bearing mice were established as mentioned previously, and 2 × 10^5^ 4T1-Luc cells were intravenously injected into the mice after 10 days postinoculation to simulate the hematogenous metastasis. The mice were randomly divided into six groups and treated as follows: G1 (saline), G2 (HRA@TT + aPD-1), G3 (HNI@D-TT + aPD-1), G4 (HNI@D-TT + aPD-1 + ATRA), G5 (HRA@D-TT), and G6 (HRA@D-TT + aPD-1). DOX formulations were administrated at a dose of 2 mg/kg on days 0, 3, 6, and 9. The aPD-1 antibody treatment was administrated intraperitoneally (100 μg per mouse for each injection) on days 1, 4, 7, and 10, which was 1 day after the HRA@D-TT administration. To establish a model of breast cancer lung metastasis, 5 × 10^5^ 4T1-Luc cells were intravenously injected into the mice on day 3 of the experiment. Tumor size and body weight were recorded daily. All the mice were euthanized after treatment, and the lung, TDLNs, and tumor tissues were sampled. Lung invasion by malignant cells was visualized by immersing harvested lungs in d-luciferin solution (15 mg/ml) for 10 min and performing bioluminescent imaging. Meanwhile, the lungs were also subjected to H&E staining to evaluate pathological changes. To analyze the immune responses, tumors collected from mice were minced into small pieces and then dissociated into single-cell suspensions using digestive enzymes. Meanwhile, single-cell suspensions from TDLNs were prepared by mechanical grinding. After filtration through a 70-μm cell strainer, the cells were stained with various fluorescent antibodies to label cell surface markers, including CD45-APC/Cy7, Gr-1-BV421, CD11b-FITC, CD11c-FITC, F4/80-PerCP/Cy5.5, CD206-APC, CD80-PE, CD86-APC, CD3-FITC, CD4-APC, CD8-PerCP/Cy5.5, CD69-BV421, Ly6G-BV421, and Ly-6C-PE. Subsequently, brefeldin A was added to the cell culture medium to block protein transport and ensure intracellular cytokine accumulation. After permeabilization using the Transcription Factor Buffer Set (BD Pharmingen), cells were stained for intracellular cytokines with IFN-γ–PE and TNF-α–APC. The populations of immune cells in tumors and lymph nodes were determined with a flow cytometer and analyzed via FlowJo. The gating strategies in this article for flow cytometry analysis of immune cell phenotypes were presented in figs. S35 to S38.

### Enhanced antitumor efficacy of HRA@D-TT and aPD-1 combination therapy in CT26 tumor models

To evaluate the combined therapeutic efficacy of HRA@D-TT and aPD-1 antibody, we established CT26 tumor models by injecting 1 × 10^6^ CT26 cells subcutaneously into BALB/c mice. Upon tumor volume reaching ~100 mm^3^, mice were randomized into four experimental groups: control (saline), aPD-1 monotherapy (aPD-1), HRA@D-TT monotherapy, and combination therapy (HRA@D-TT + aPD-1). HRA@D-TT was administered intravenously on days 0, 3, 6, and 9, while aPD-1 was administered intraperitoneally on days 1, 4, 7, and 10. Tumor dimensions were measured daily throughout the experimental period. The study was terminated on day 12, and the mice were euthanized for tissue collection, including tumors, TDLNs, and spleens. Harvested tumor specimens were enzymatically dissociated into single-cell suspensions using a multi-enzyme digestion solution. Subsequently, cells were labeled with fluorochrome-conjugated antibodies, and immune cell populations were characterized quantitatively via flow cytometric analysis.

### Comparative study of the antitumor efficacy of different nano-formulations

The preparative DA NPs were fabricated by dissolving ATRA (8 mg), DOX (2 mg), and PEG-b-PLA (100 mg) in 2 ml of mixed organic solvents (chloroform:DMSO = 3:1, v/v), which were combined with water and emulsified via probe sonication in an ice bath. The resulting products underwent vacuum evaporation, dialysis, and ultrafiltration to yield DA NPs. For DiR-labeled PLA nanoparticles (DD NPs), DD NPs were synthesized following an identical methodology, substituting ATRA with DiR (3 mg). For animal studies, a 4T1 tumor model was established in BALB/c mice according to previously described protocols. Once tumor volumes reached ~100 mm^3^, mice were randomly allocated into five experimental groups (G1, saline; G2, Doxil; G3, HNI@D-TT; G4, DA NPs; and G5, HRA@D-TT) and administered corresponding formulations intravenously (DOX equivalent dose of 2 mg/kg) at 3-day intervals. One cohort (*n* = 5) was monitored for tumor progression, with tumor tissues harvested on day 14 for flow cytometric analysis. A separate cohort (*n* = 5) was evaluated for survival outcomes. To investigate intratumoral distribution patterns of various therapeutic agents, DD NPs, HDiR@D-TT, Doxil, and HNI@D-TT were administered intravenously to tumor-bearing mice. Tumor specimens were collected 24 hours postinjection and sectioned for CLSM examination. Tissues from DD NPs and HDiR@D-TT treatment groups were stained for HIF-1α, while samples from Doxil and HNI@D-TT groups were immunolabeled with CD31 antibodies.

### Statistical analysis

All results are presented as means ± SD. Comparisons between two groups were conducted using the two-tailed Student’s *t* test, while multiple group comparisons were analyzed using one-way analysis of variance (ANOVA) followed by Tukey’s post hoc tests. Statistical significance was indicated by *P < 0.05, **P < 0.01, ***P < 0.001, and ****P < 0.0001. All analyses were performed using GraphPad Prism 8 and Microsoft Excel 2019 software.
