# Supplementary material for: Pulsatile sequential drug release system for cascade tumor deep penetration and differentiation therapy to enhance chemoimmunotherapy
Source: Sci Adv. 2025 Sep 3;11(36):eadr8001. doi: 10.1126/sciadv.adr8001 (PMC12407085; doi:10.1126/sciadv.adr8001)
Supplement: Supplementary file 1 — Figs. S1 to S38 Table S1 [file sciadv.adr8001_sm.pdf]

Science Advances
AAAS

Supplementary Materials for

Pulsatile sequential drug release system for cascade tumor deep penetration and differentiation therapy to enhance chemoimmunotherapy

Fengxiang Liu *et al.*

Corresponding author: Kaiyuan Wang, wangkaiyuan@hotmail.com; Jin Sun, sunjin@syphu.edu.cn

Sci. Adv. **11**, eadr8001 (2025)
DOI: 10.1126/sciadv.adr8001

This PDF file includes:

Figs. S1 to S38
Table S1

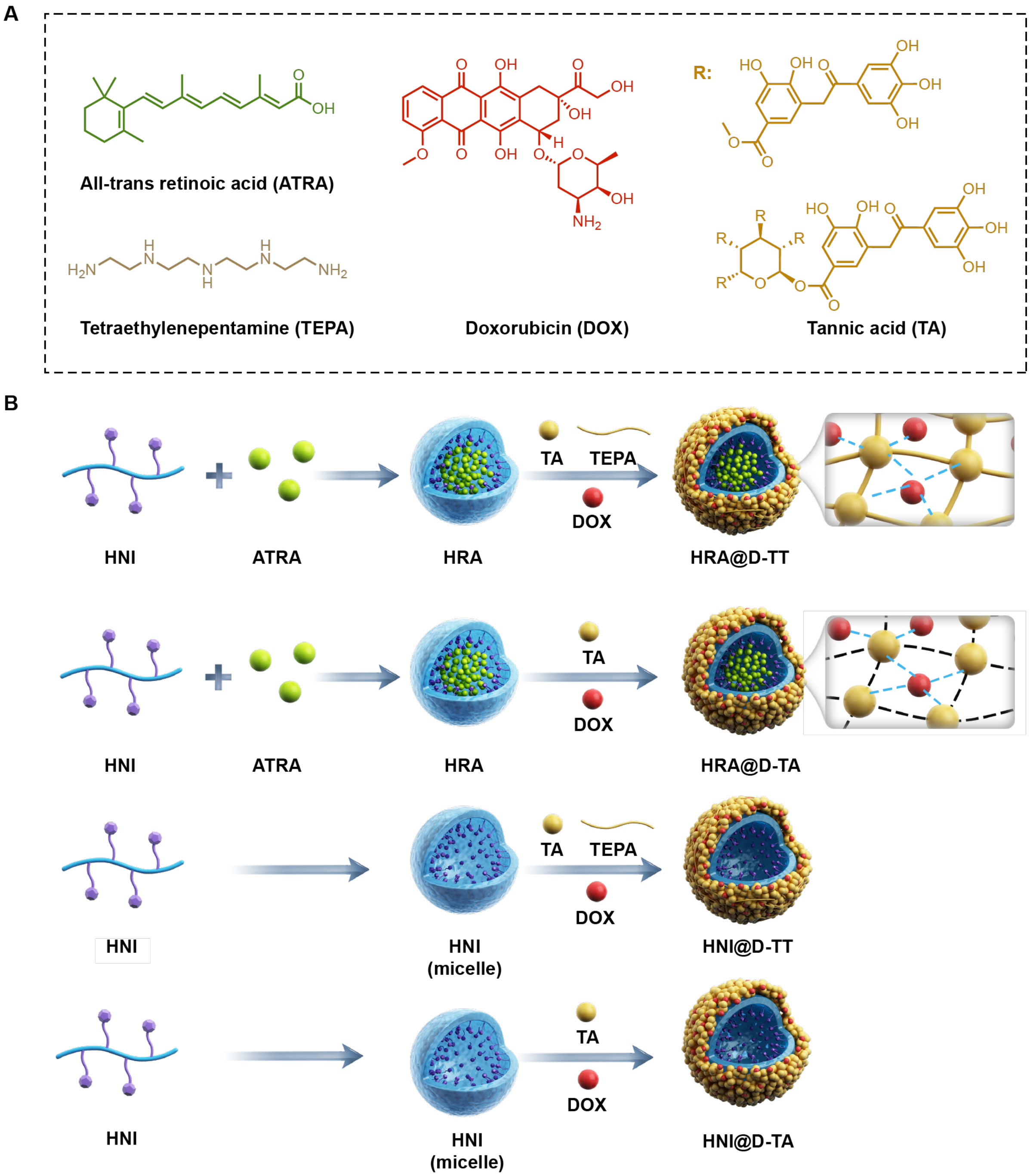

Fig. S1. (A) Structures and abbreviations of the main chemical substances mentioned in the text. **(B)** Compositions and abbreviations of the main nanoplatforms mentioned in the text.

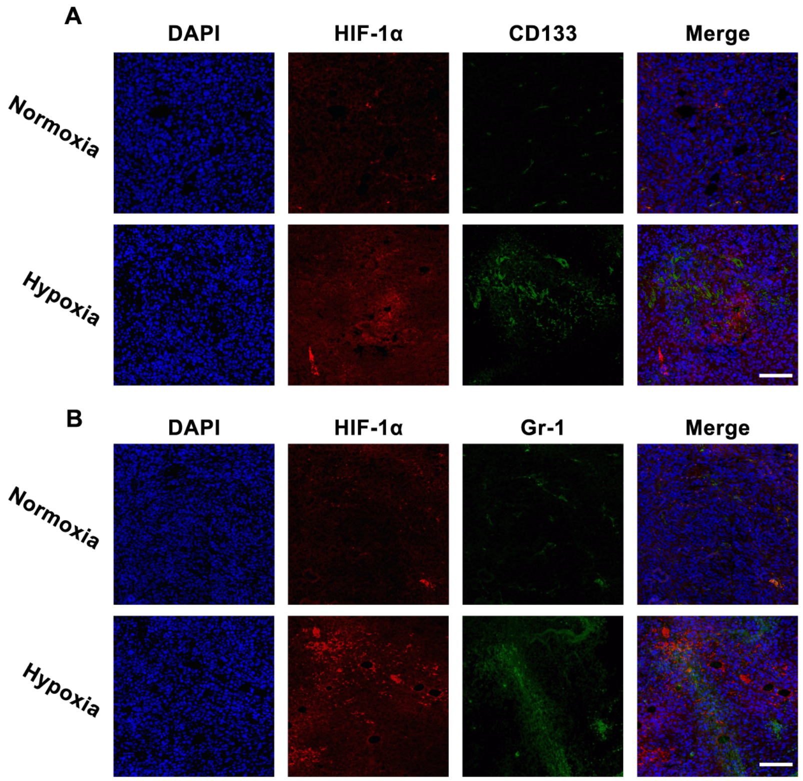

Fig. S2. Immunofluorescence staining for investigating the heterogeneous distribution. (A) CSCs (CD133) and **(B)** MDSCs (Gr-1) in the hypoxic (HIF-1α) and normoxic regions of tumors. Scale bar: 200 μm.

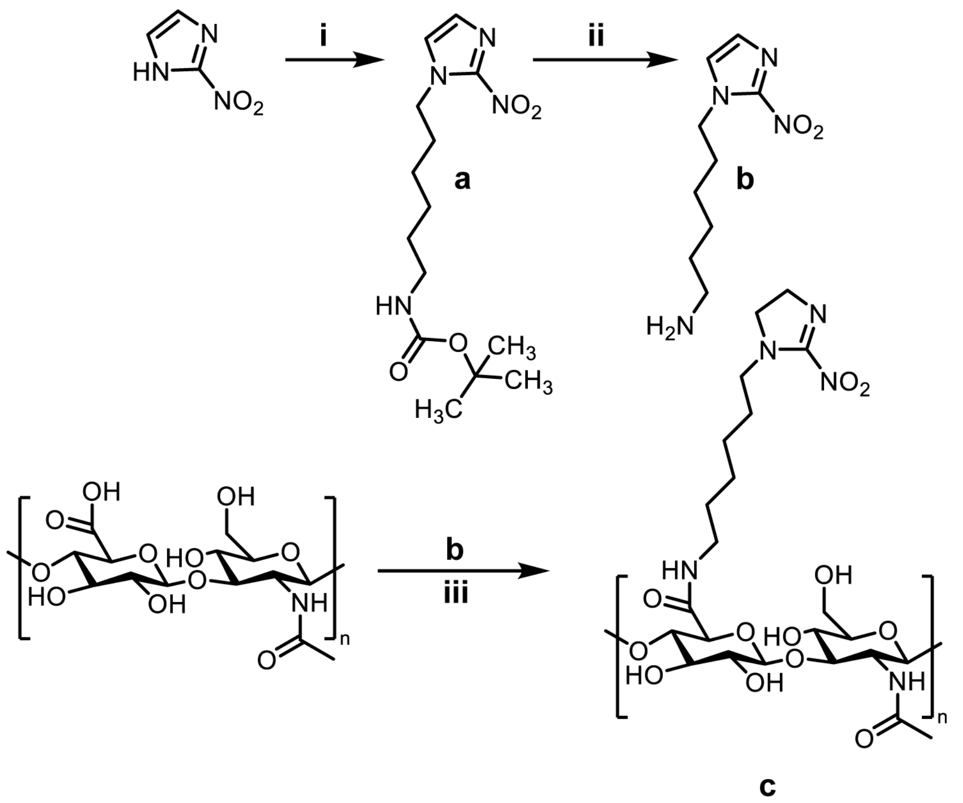

Fig. S3. Synthetic routes of nitroimidazole-modified hyaluronic acid. Reagents and conditions: (i) K_2CO_3, 6-(Boc-amino) hexyl bromide, DMF, 80°C; (ii) CH_3OH-HCl, 25°C; (iii) EDC, NHS, H_2O, 25°C.

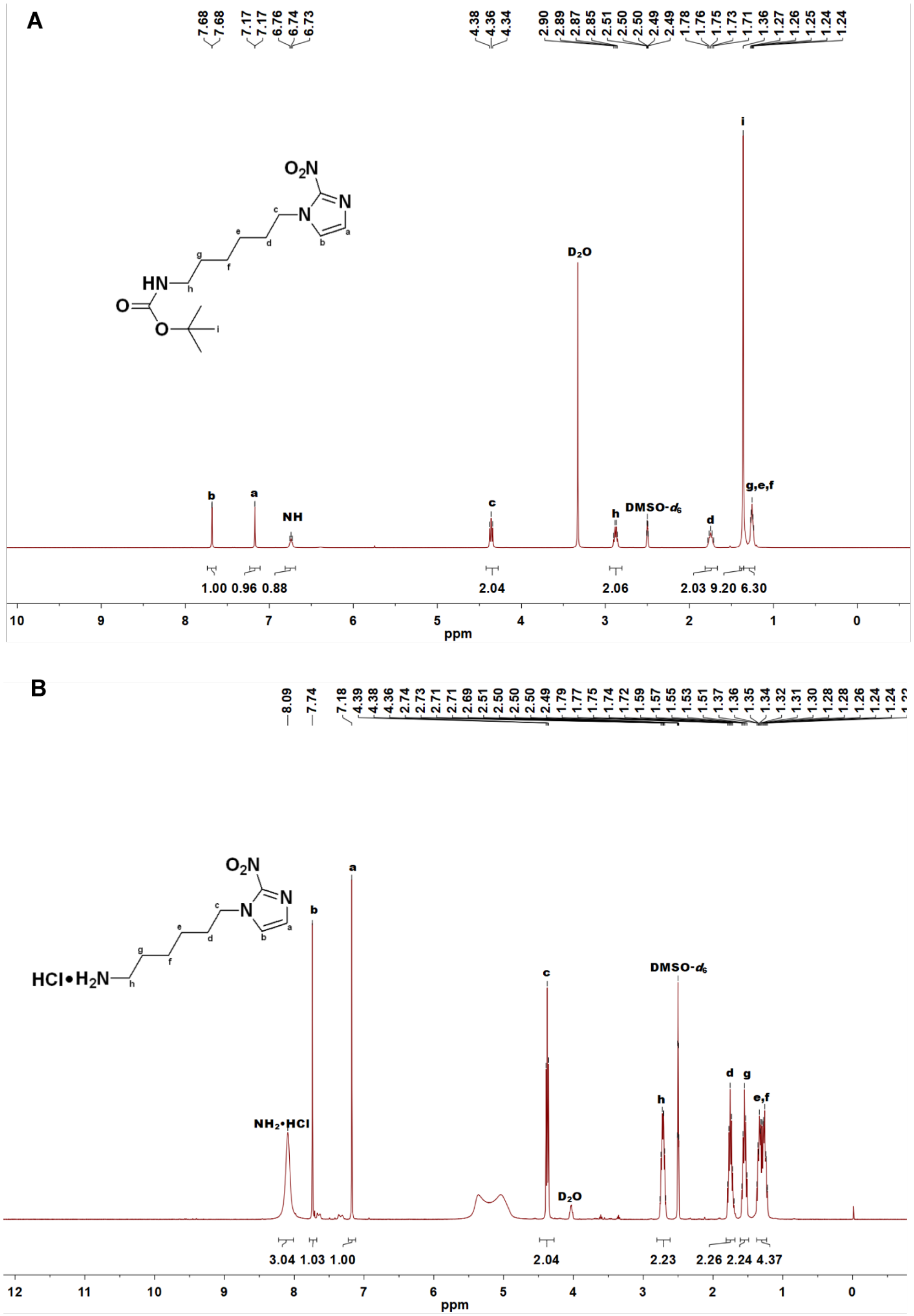

Fig. S4. ^{1}H NMR spectrum of (A) Boc-protected NI-NH and (B) NI-NH.

Boc-protected NI-NH: ^{1}H NMR (400 MHz, DMSO-d6) δ 7.68 (d, J = 1.0 Hz, 1H, H-b), 7.17 (d, J = 1.0 Hz, 1H, H-a), 6.74 (t, J = 5.8 Hz, 1H, NH), 4.36 (t, J = 7.3 Hz, 2H, H-c), 2.88 (q, J = 6.5 Hz, 2H, H-h), 1.75 (t, J = 7.2 Hz, 2H, H-d), 1.36 (s, 9H, H-i), 1.35-1.22 (m, 6H, H-g,e,f).

NI-NH: ^{1}H NMR (400 MHz, DMSO-d6) δ 8.09 (s, 3H, NH2•HCl), 7.74 (s, 1H, H-b), 7.18 (s, 1H, H-a), 4.38 (t, J = 7.3 Hz, 2H, H-c), 2.80-2.61 (m, 2H, H-h), 1.74 (h, J = 7.6 Hz, 2H, H-d), 1.55 (p, J = 7.3 Hz, 2H, H-g), 1.37-1.23 (m, 4H, H-e,f).

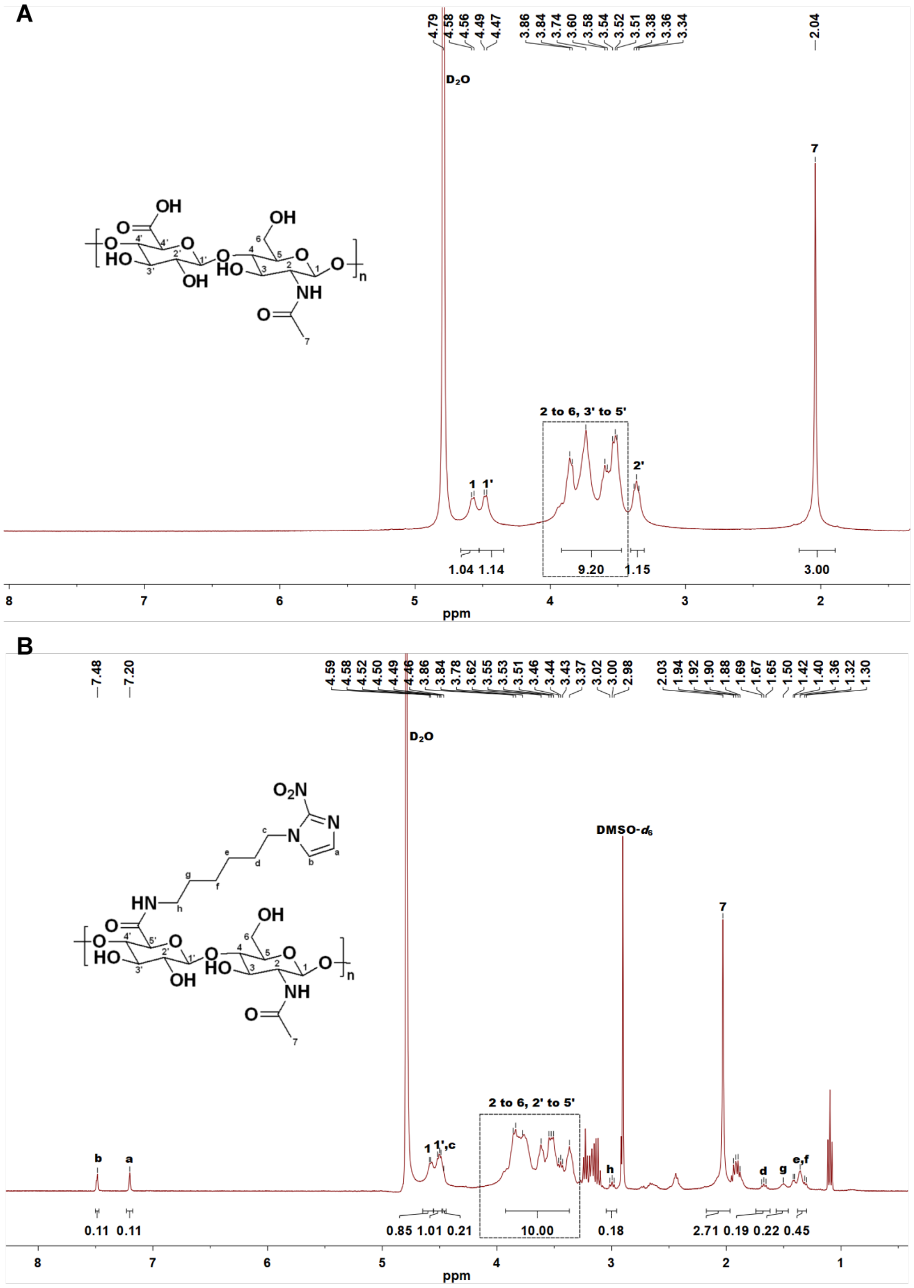

Fig. S5. ^{1}H NMR spectrum of (A) HA and (B) HNI.

HA: ^{1}H NMR (400 MHz, D2O) δ 4.57 (d, J = 8.0 Hz, 1H, H-1), 4.48 (d, J = 7.2 Hz, 1H, H-1’), 3.95-3.43 (m, 9H, H-2 to H-6, H-3’ to H-5’), 3.36 (t, J = 5.9 Hz, 1H, H-2’), 2.04 (s, 3H, H-7).

HNI: ^{1}H NMR (400 MHz, D2O) δ 7.48 (s, 0.11H, H-b), 7.20 (s, 0.11H, H-a), 4.65-4.55 (m, 1H, H-1), 4.55 – 4.48 (m, 1H, H-1’), 4.46 (s, 0.21H, H-c), 3.93-3.37 (m, 10H, H-2 to H-6, H-2’ to H-5’), 3.00 (t, J = 7.6 Hz, 0.18H, H-h), 2.03 (s, 3H, H-7), 1.74-1.62 (m, 0.19H, H-d), 1.50 (s, 0.22H, H-g), 1.34 (d, J = 15.6 Hz, 0.45H, H-e,f).

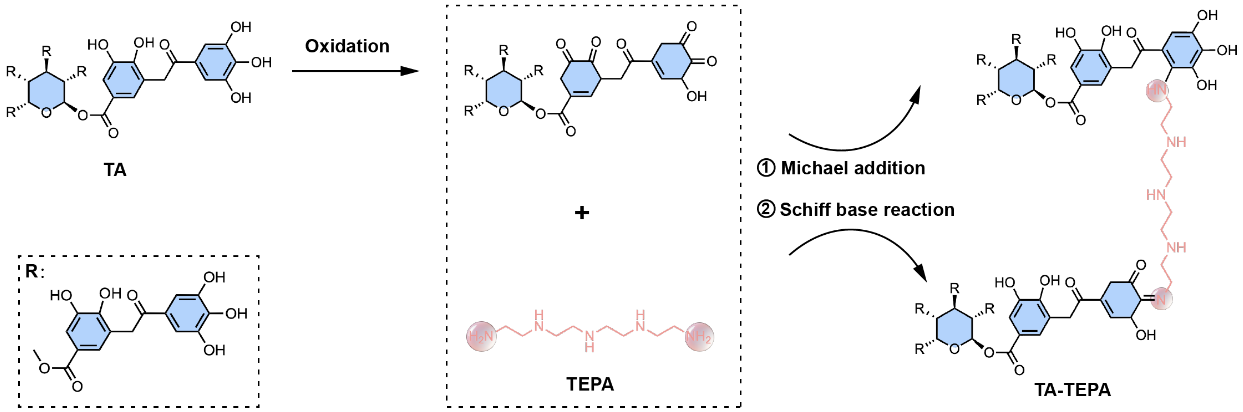

Fig. S6. Schematic diagram of the reaction mechanism between TA and TEPA.

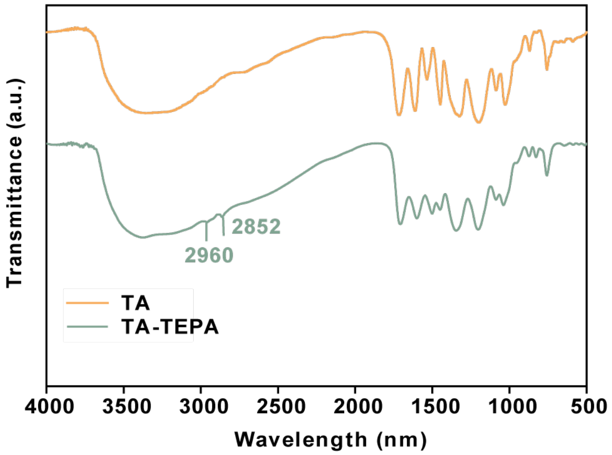

Fig. S7. The FTIR spectrum of TA and TA-TEPA polymer.

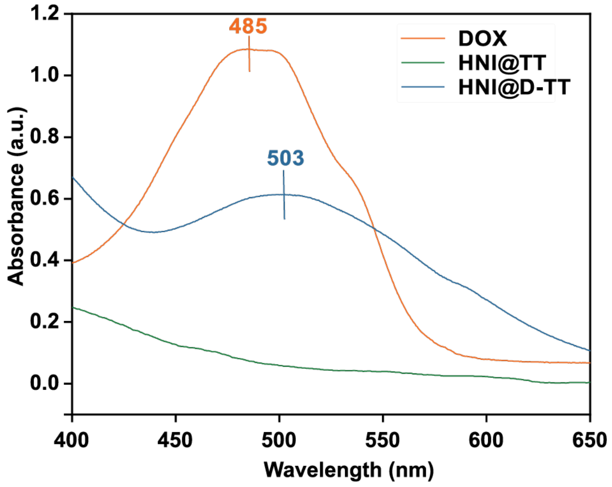

Fig. S8. The UV spectra of DOX, HNI@TT, and HNI@D-TT.

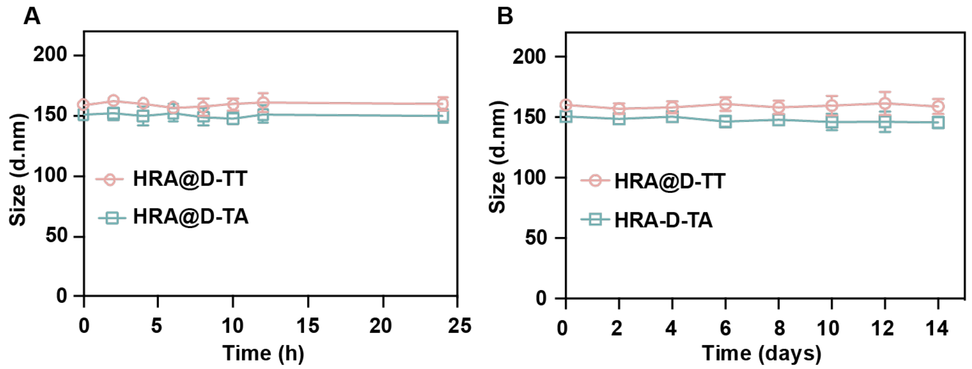

Fig. S9. Stability studies. (A) Stability of HRA@D-TT and HRA@D-TA incubated in PBS with 10% FBS at 37 °C for 24 h (n=3). **(B)** Stability of HRA@D-TT and HRA@D-TA stored at 4 °C for 14 days (n=3). Data are presented as mean ± SD.

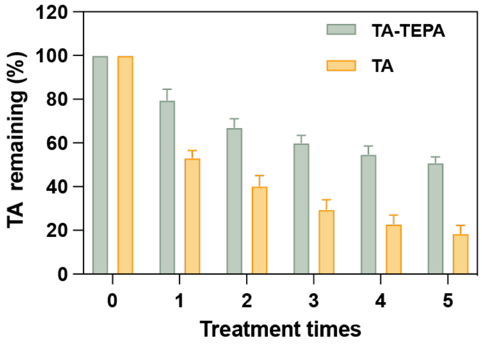

Fig. S10. Residual TA and TA-TEPA layer coated on the surface of nanoparticles after treatments (n=3). Data are presented as mean ± SD.

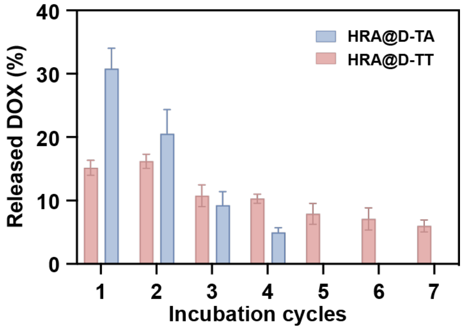

Fig. S11. The released DOX during each incubation cycle from HRA@D-TT and HRA@D-TA (n=3). Data are presented as mean ± SD.

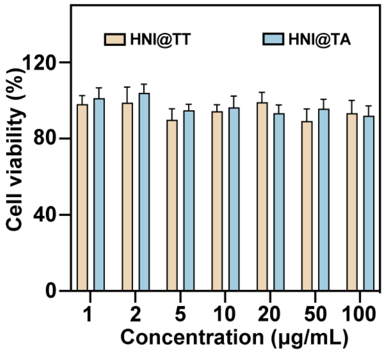

Fig. S12. The 48 h biocompatibility study of HNI@TT and HNI@TA (n=3). Data are presented as mean ± SD.

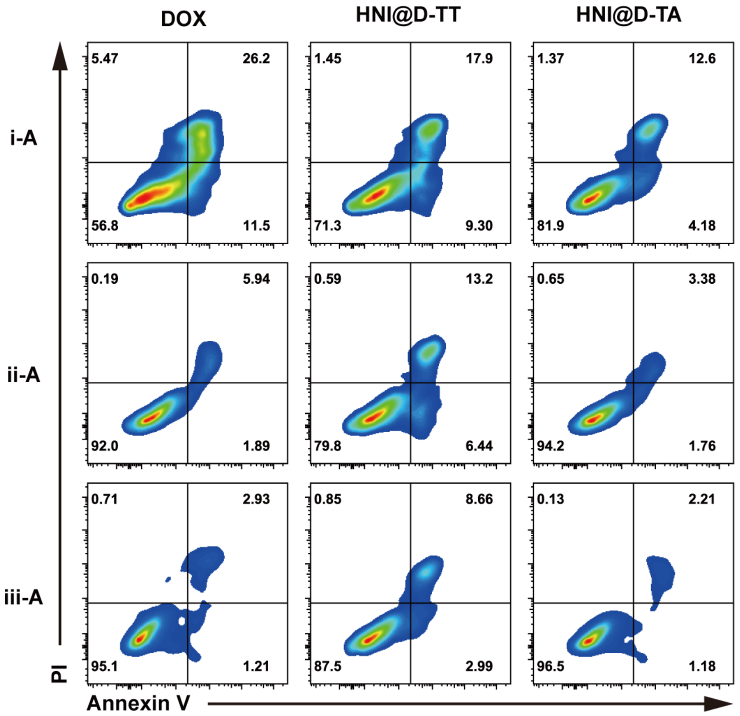

Fig. S13. Representative flow cytometry images of the apoptotic rates of the cells in wells (i), (ii), and (iii).

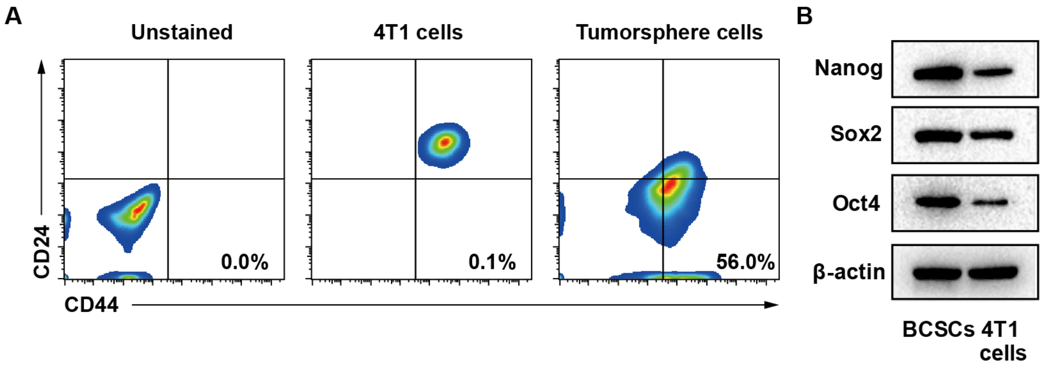

Fig. S14. Characterization of CSC-rich tumorsphere cells. (A) Representative flow cytometry images of CD44^{+}/CD24^{-} cells in adherent 4T1 cells and tumorsphere cells. **(B)** Western blot characterization of the expression levels of Nanog, Sox2, and Oct4 in adherent 4T1 cells and BCSCs.

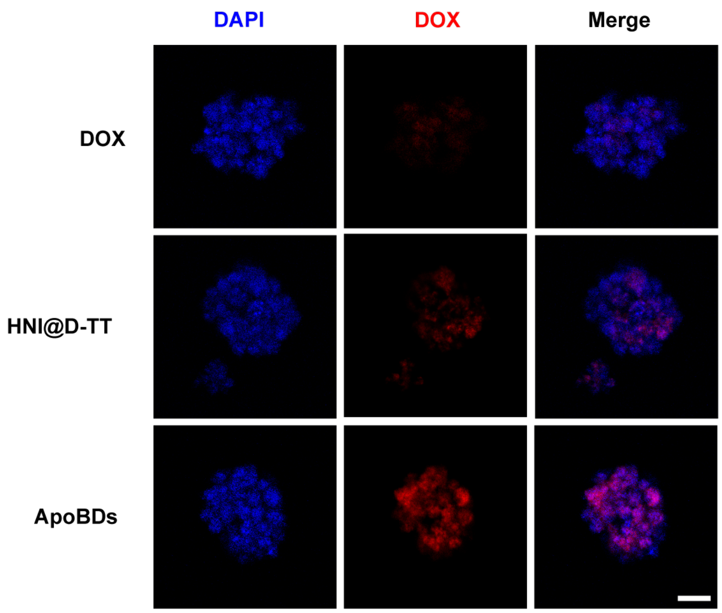

Fig. S15. CLSM images of tumorsphere cells treated for 4 h. Scale bar, 20 μm.

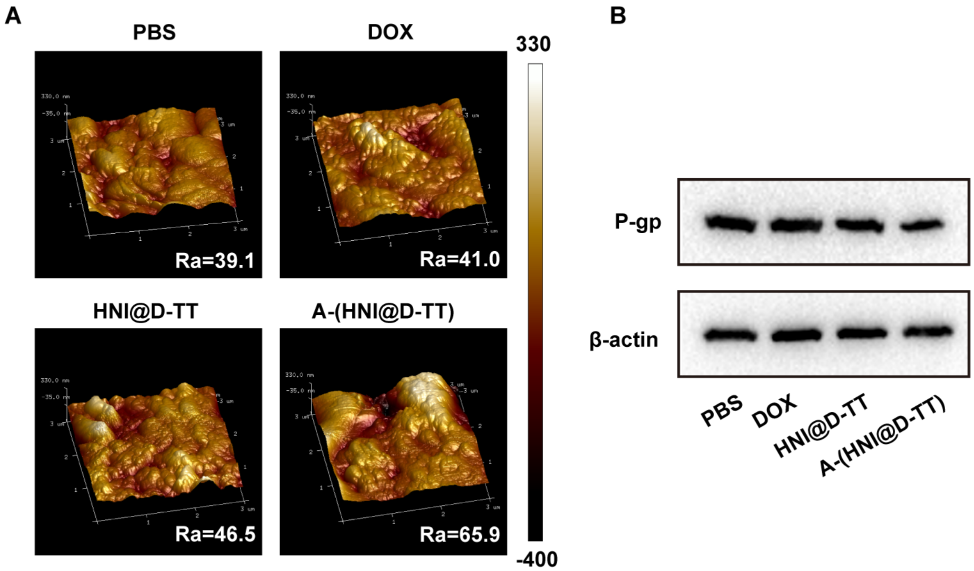

Fig. S16. The investigation of the interaction of cells with HRA@D-TT. (A) Differences in surface roughness (Ra) of tumorsphere cells with different treatments observed via AFM. **(B)** The P-gp expression analyzed by Western blot under different treatments.

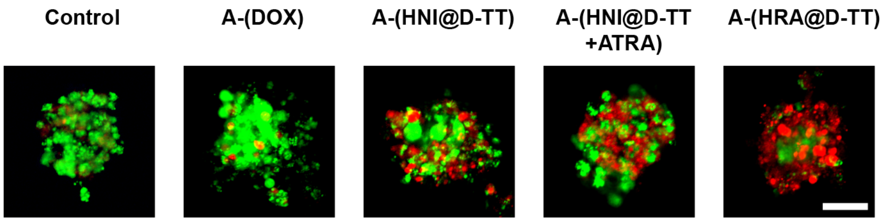

Fig. S17. Calcein-AM/PI staining images of tumorspheres after various treatments in hypoxia. Red, dead cells; Green, live cells. Scale bar, 50 μm.

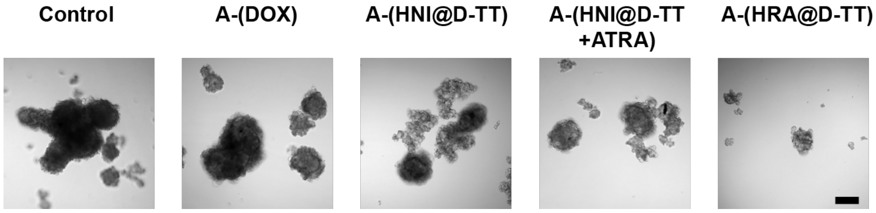

Fig. S18. The representative images of the tumorsphere formation assay. Scale bar, 50 μm.

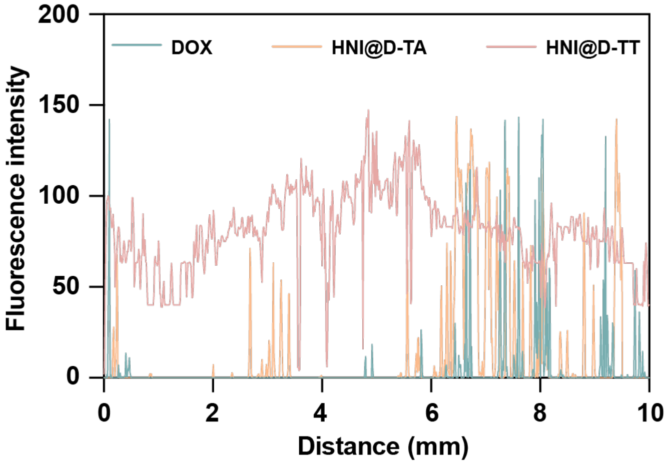

Fig. S19. Semi-quantitative analysis of tumor sections in Fig. 6D.

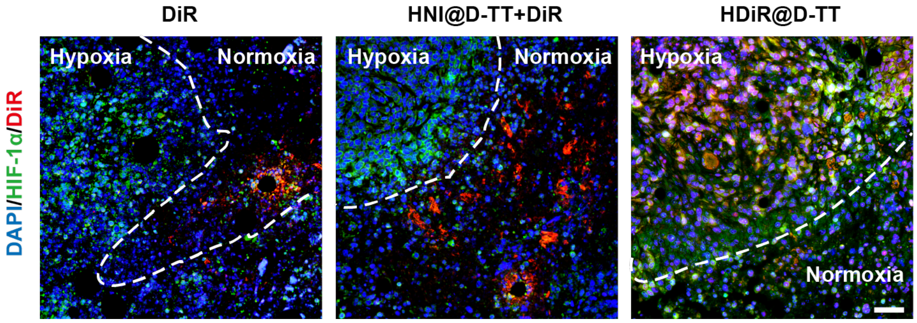

Fig. S20. **CLSM images of tumor sections after intravenous injection of DiR, HNI@D-TT+DiR, and HDiR@D-TT at 24 h.** Hoechst 33342, blue; DiR, red; HIF-1α, green. Scale bar represents 50 μm.

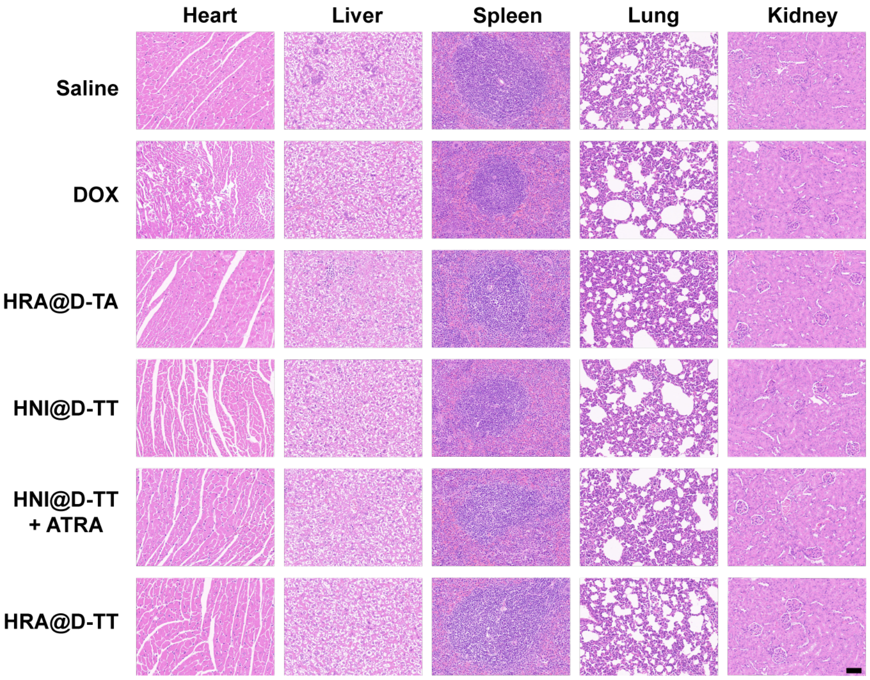

Fig. S21. H&E staining images of the major organs of 4T1 tumor-bearing mice at the end of treatment. Scale bar, 50 μm.

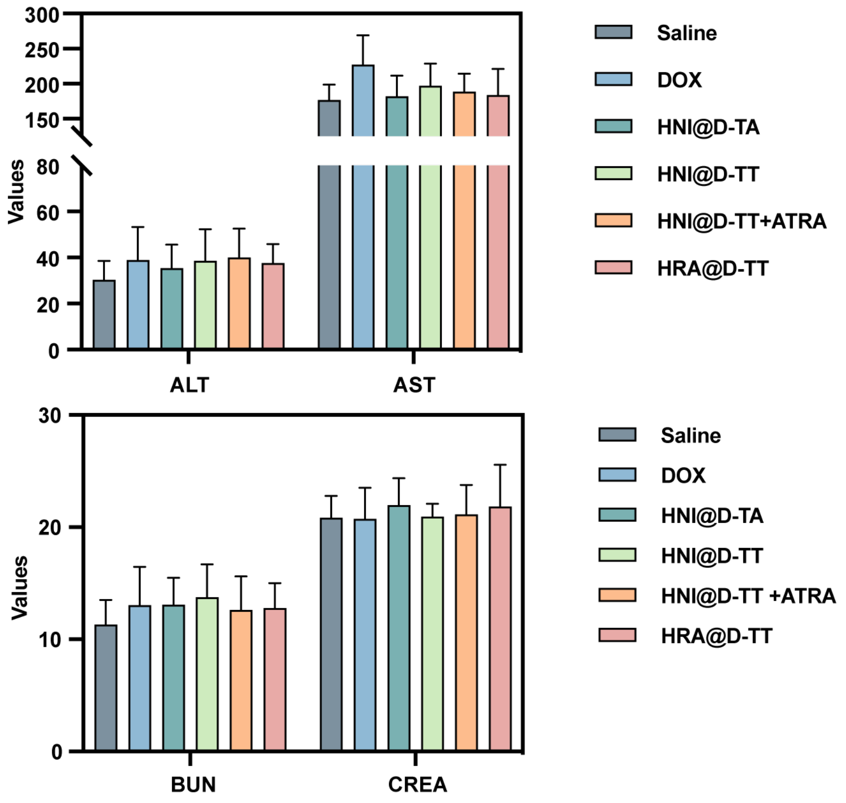

Fig. S22. Hematological parameters (ALT, AST, BUN, and CREA) of mice after various treatments (n=3 mice). Data are presented as the mean ± SD.

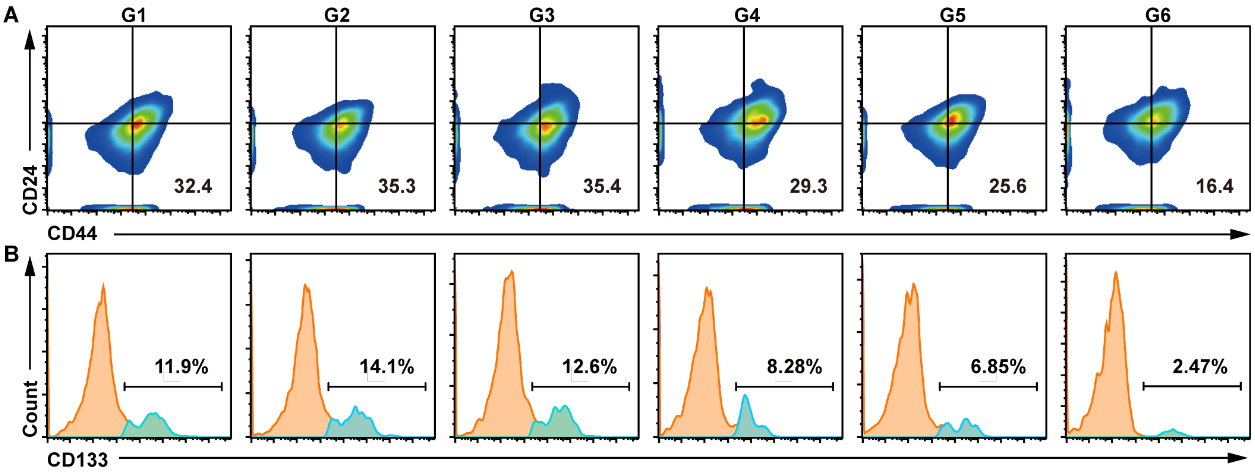

Fig. S23. Representative flow cytometry images of CSCs in tumors. **(A)** Representative flow cytometry images of $CD44^+/CD24^-$ cells in tumor tissues. **(B)** Representative flow cytometry images of $CD133^+$ cells in tumor tissues.

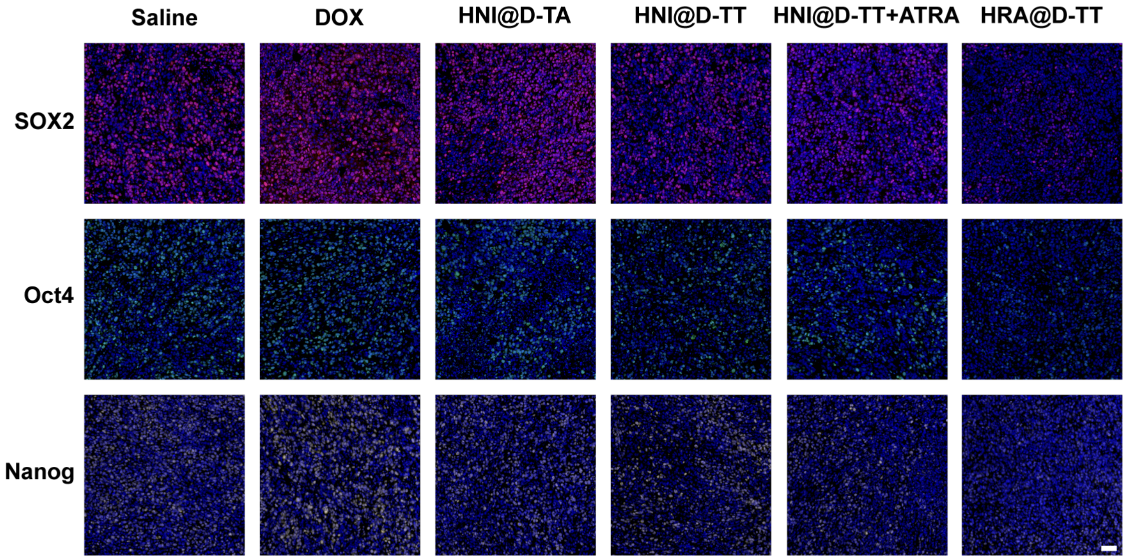

Fig. S24. CLSM images of tumor sections stained with stemness-related pluripotency factors. Scale bar, 50 μm.

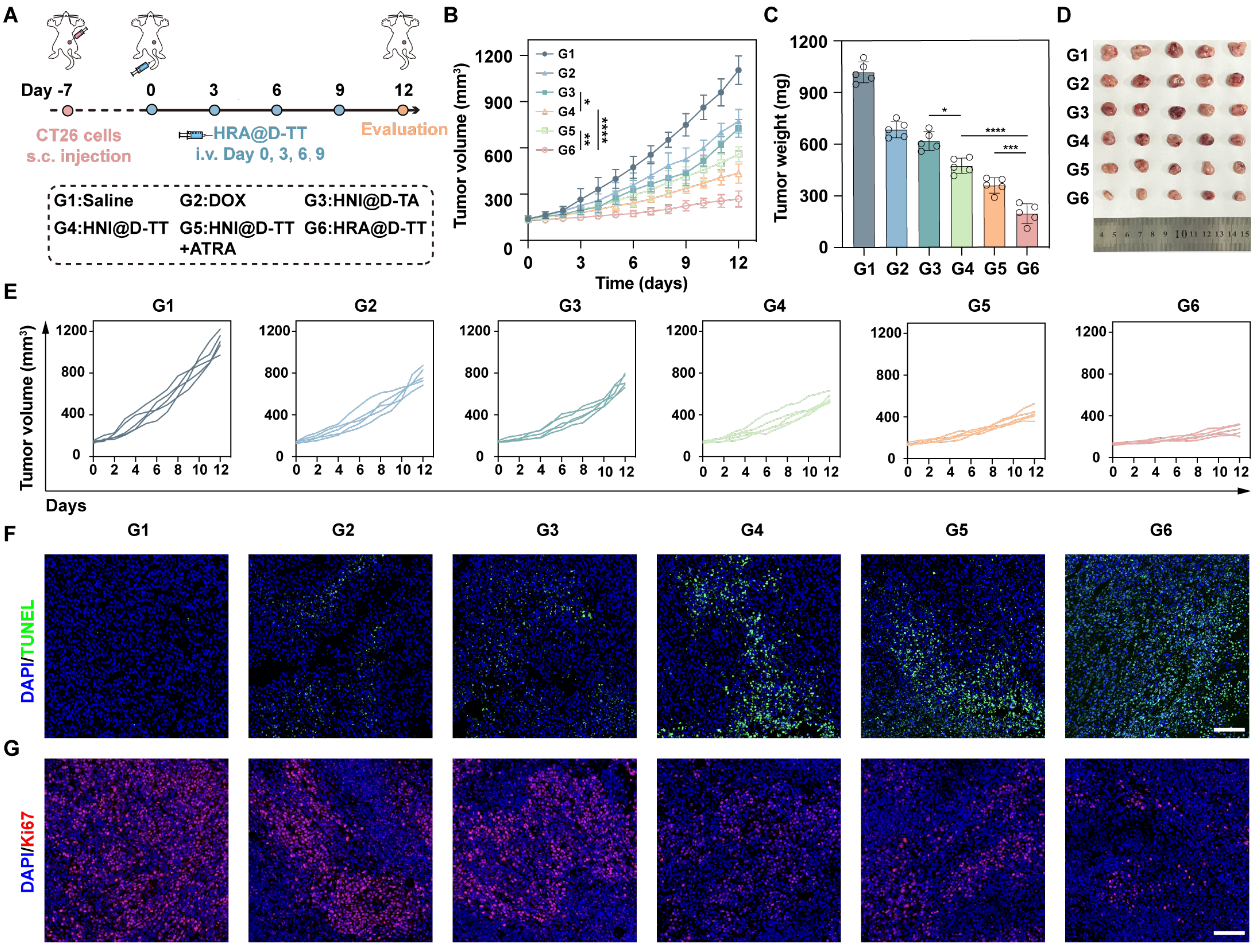

Fig. S25. Anti-tumor efficacy in CT26 tumor model. (A) Schema showing the therapeutic interventions in CT26 tumor-bearing mice. **(B)** Tumor volume growth profiles of CT26 xenografts (n=5 mice). **(C)** Tumor weight after different treatments (n=5 mice). **(D)** Tumor photos after the final treatment (n=5 mice). **(E)** Individual volume growth profiles of CT26 xenografts (n=5 mice). **(F)** TUNEL and **(G)** Ki67 staining images of tumor sections after different treatments. Scale bar represents 100 μm. Data are represented as mean ± SD and statistical significance was analyzed through one-way analysis of variance (ANOVA). $*p < 0.05$, $**p < 0.01$, $***p < 0.001$, $****p < 0.0001$; n.s. indicates no significance.

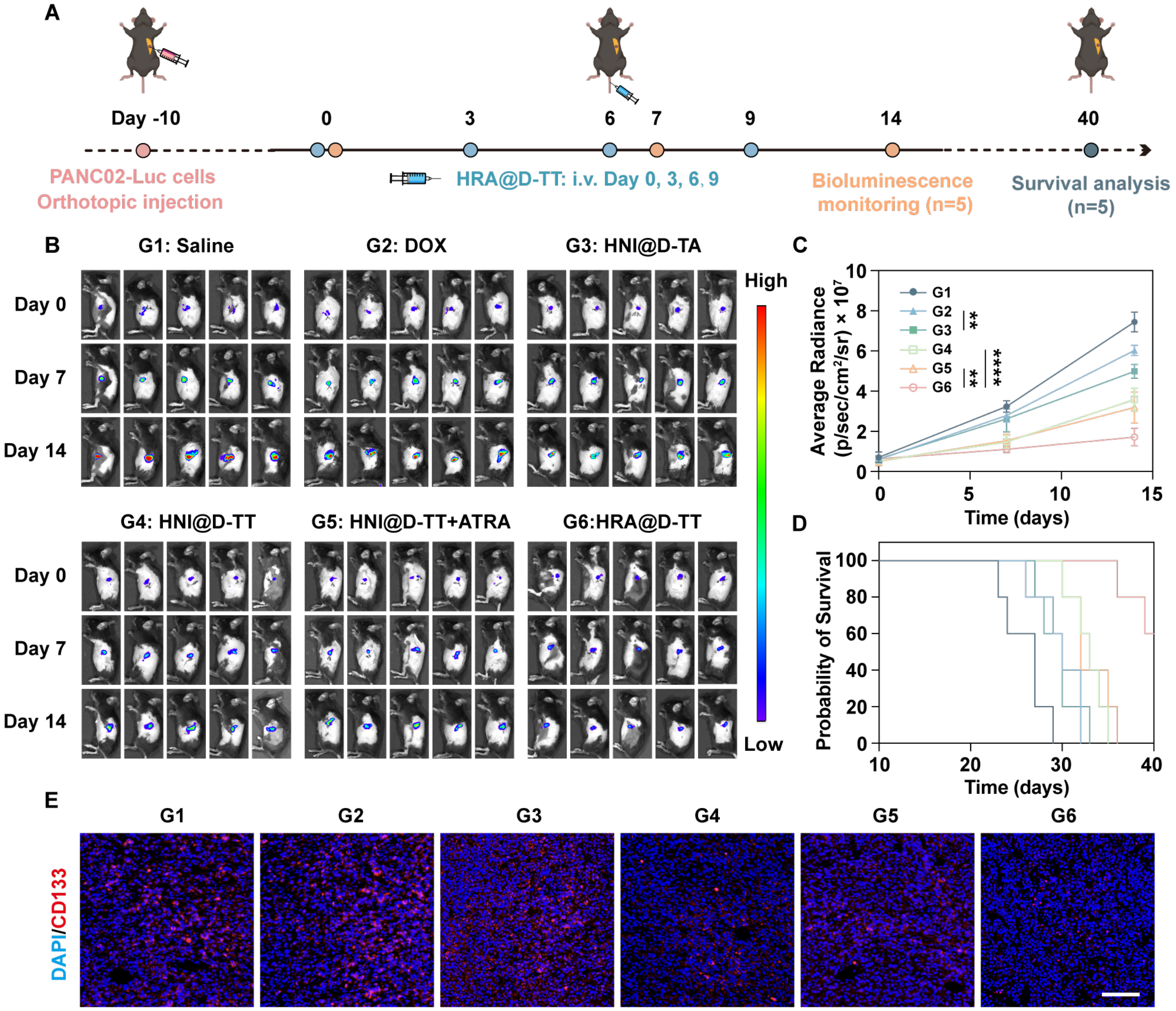

Fig. S26. Anti-tumor efficacy in orthotopic PANC02-Luc pancreatic tumors. (A) Timeline for the therapeutic interventions of orthotopic PANC02-Luc tumor model in C57BL/6 mice. **(B)** Bioluminescence images, **(C)** bioluminescence intensities, and **(D)** survival curves of mice in different groups (n=5 mice). **(E)** CLSM images of CD133 expression in PANC02-Luc tumor sections after different treatments. Scale bar represents 100 μm. Data are represented as mean ± SD and statistical significance was analyzed through one-way analysis of variance (ANOVA). $^*p < 0.05$, $^{**}p < 0.01$, $^{***}p < 0.001$, $^{****}p < 0.0001$; n.s. indicates no significance.

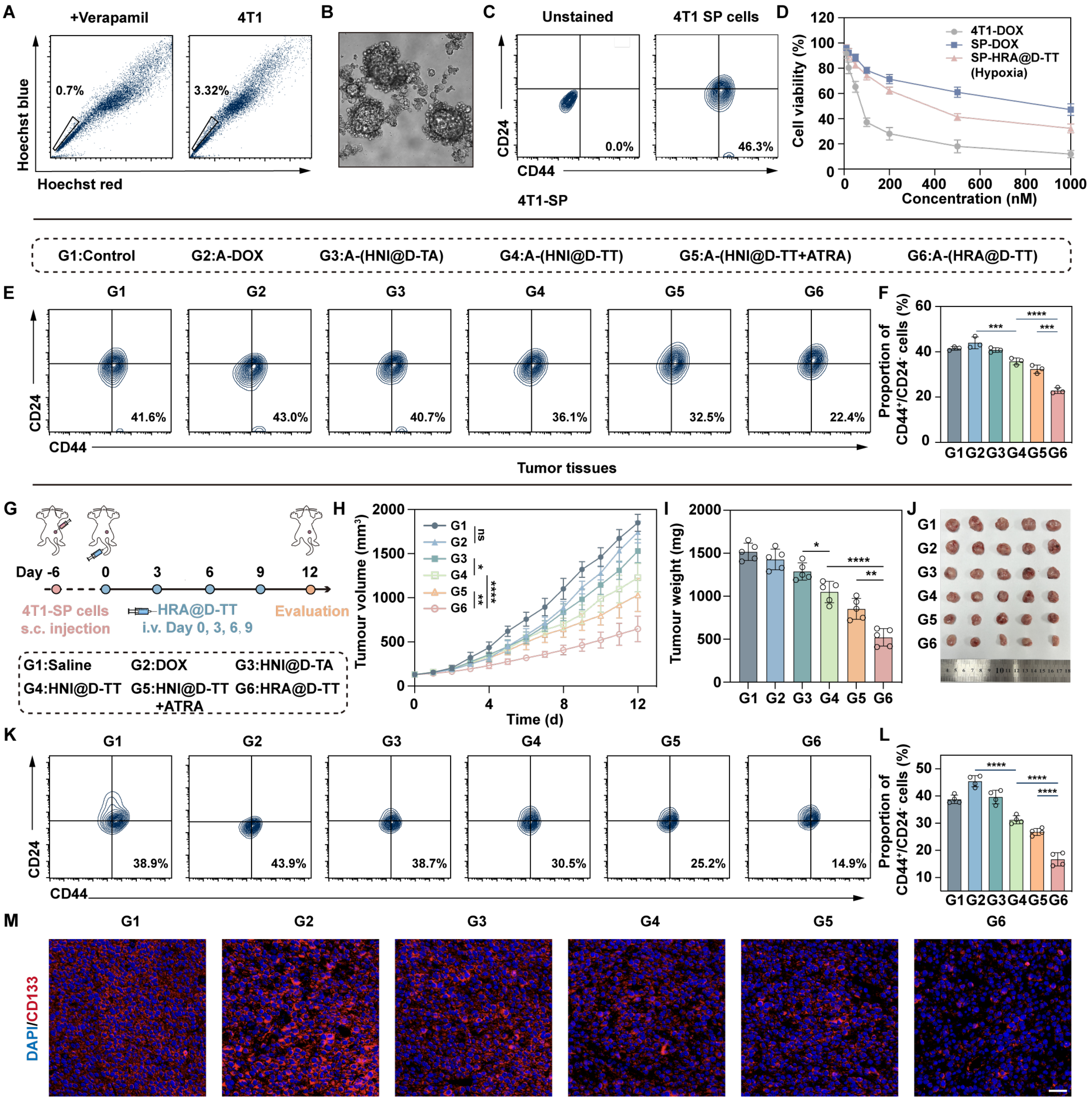

Fig. S27. **HRA@D-TT potentiates chemotherapy through CSC differentiation in 4T1-SP cells.** **(A)** Representative images of SP cell isolation in 4T1 breast cancer cells. **(B)** The photo of the tumourspheres formed by 4T1 SP cells after 7 days of culture. **(C)** Representative flow cytometry images of CD44^{+}/CD24^{-} CSCs in 4T1-SP cells after 7 days of culture. **(D)** Viabilities of 4T1-SP cells after various treatments for 48 h (n = 3). **(E)** Representative flow cytometry images and **(F)** qualification of CD44^{+}/CD24^{-} CSCs in 4T1-SP tumorspheres after treatments with different ApoBDs for 48 h under hypoxia (n = 3). **(G)** Protocol of the antitumor efficacy study in BALB/C mice. **(H)** Tumor volume, **(I)** tumor weight, and **(J)** tumor images after various treatments (n=5 mice). **(K)** Representative flow cytometry images and **(L)** qualification of CD44^{+}/CD24^{-} CSCs in tumor tissues (n=5 mice). **(M)** CLSM images of CD133 expression in tumor sections after different treatments. Scale bar represents 40 μm. Data are represented as mean

± SD and statistical significance was analyzed through one-way analysis of variance (ANOVA). $^{*}p < 0.05$, $^{**}p < 0.01$, $^{***}p < 0.001$, $^{****}p < 0.0001$; n.s. indicates no significance

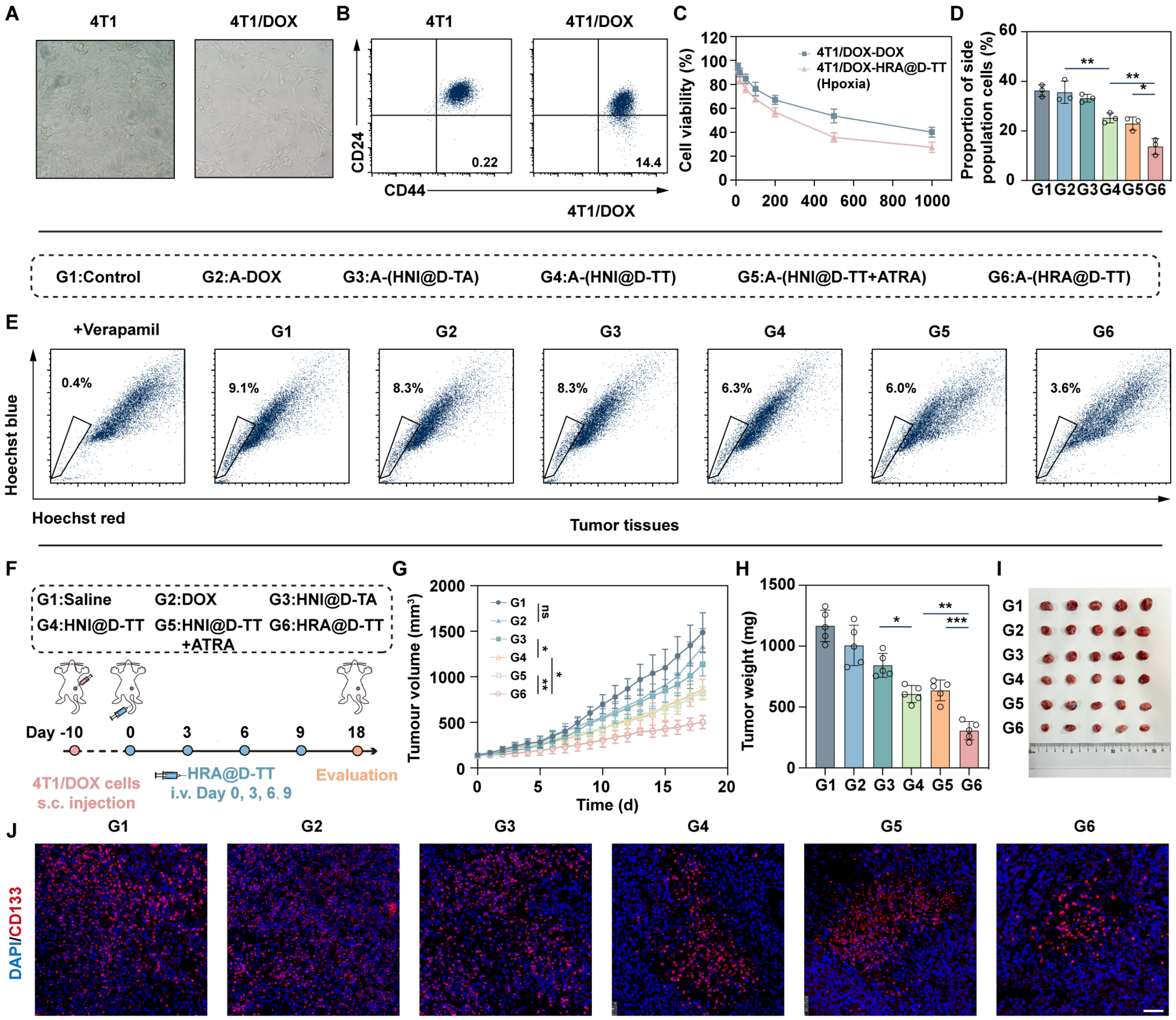

Fig. S28. **HRA@D-TT enhanced DOX sensitivity in drug-resistant cells.** **(A)** The images of 4T1 and 4T1/DOX cells. **(B)** Representative flow cytometry images of $CD44^+/CD24^-$ cells in 4T1 and 4T1/DOX cells. **(C)** Cell viabilities of 4T1-SP cells after various treatments for 48 h (n=3). **(D)** Qualification and **(E)** representative flow cytometry images of SP cells in 4T1/DOX tumorsphere cells after treatments with different ApoBDs for 48 h under hypoxia (n=3). **(F)** Schema depicting the 4T1/DOX tumor-bearing model construction and treatment schedule. **(G)** Tumor volume, **(H)** tumor weight, and **(I)** tumor images after various treatments (n=5 mice). **(J)** CLSM images of tumor sections after different treatments. Scale bar represents 100 μm. Data are represented as mean ± SD and statistical significance was analyzed through one-way analysis of variance (ANOVA). $^*p < 0.05$, $^{**}p < 0.01$, $^{***}p < 0.001$, $^{****}p < 0.0001$; n.s. indicates no significance.

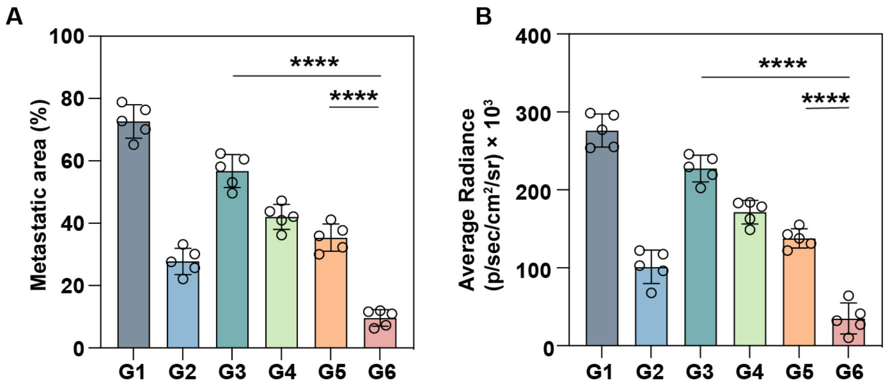

Fig. S29. Quantification results of the lung metastasis. **(A)** Quantification results for metastasis area percentages of H&E staining of lung sections (n=5 mice). **(B)** Quantification results of the bioluminescent radiance of lungs after the last treatment (n=5 mice). Data are presented as the mean ± SD.

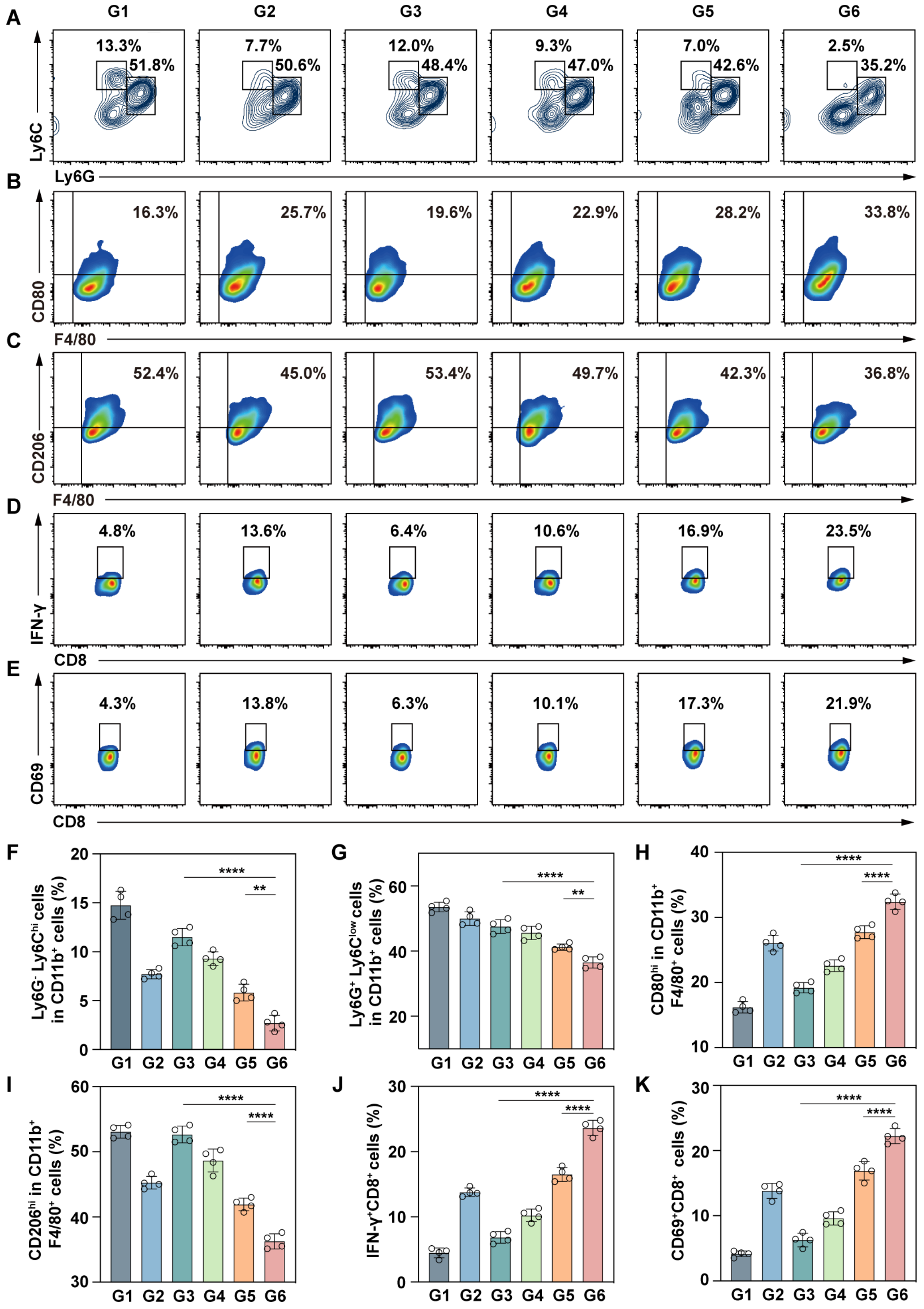

Fig. S30. Flow cytometry measurement of immune cells in tumors of Fig. 8. **(A)** CD11b^{+}Ly6G^{-} Ly6C^{hi} M-MDSCs or CD11b^{+}Ly6G^{+}Ly6C^{low} PMN-MDSCs, **(B)** CD11b^{+}F4/80^{+}CD80^{hi} M1-like macrophages, **(C)** CD11b^{+}F4/80^{+}CD206^{hi} M2-like macrophages, **(D)** IFN-γ^{+}CD8^{+} T cells, and **(E)** CD69^{+}CD8^{+} T cells in tumor. Quantification analysis of **(F)** M-MDSCs, **(G)** PMN-MDSCs in **(A)** (n=4 mice). **(H)** Relative quantification in **(B)** (n=4 mice). **(I)** Relative quantification in **(C)** (n=4 mice). **(J)** Relative quantification in **(D)** (n=4 mice). **(K)** Relative quantification in **(E)** (n=4 mice).

Data are represented as mean ± SD and statistical significance was analyzed through one-way analysis of variance (ANOVA). $*p < 0.05$, $**p < 0.01$, $***p < 0.001$, $****p < 0.0001$; n.s. indicates no significance.

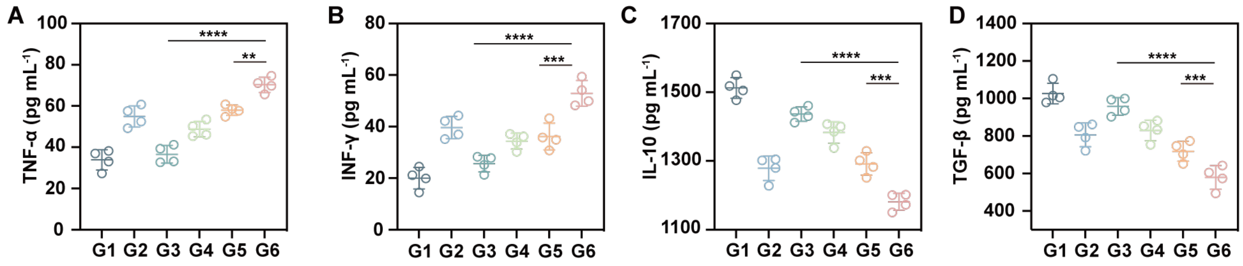

Fig. S31. The intratumor secretion of representative cytokines (A) TNF-α, (B) IFN-γ, (C) IL-10, and (D) TGF-β (n = 4 mice). Data are represented as mean ± SD.

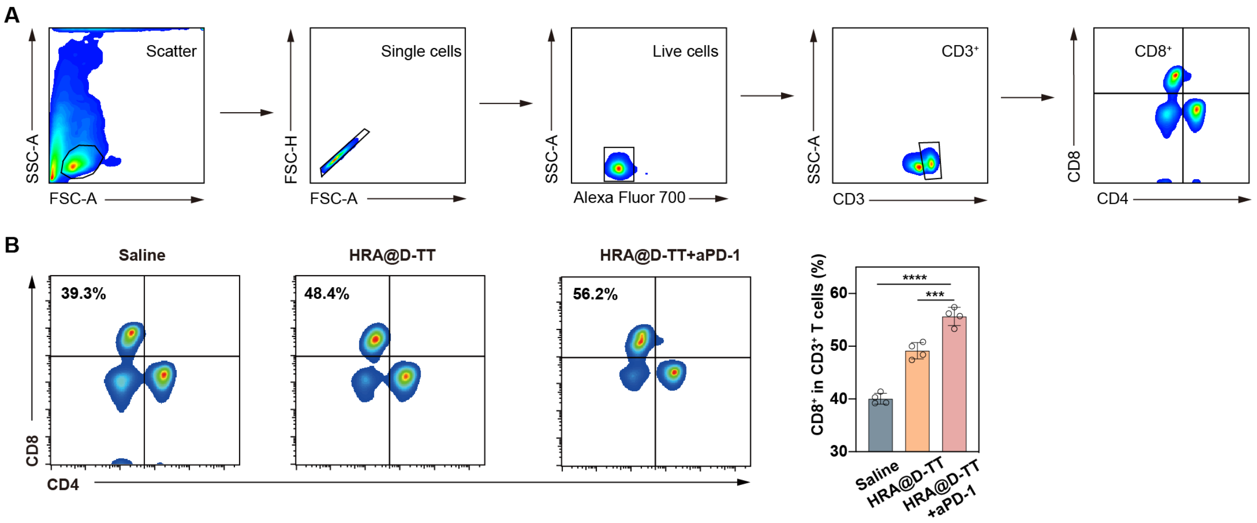

Fig. S32. Flow cytometry measurement of $CD8^+$ T cells in blood. (A) Gating strategy for identifying $CD8^+$ T cells in blood. **(B)** Representative flow cytometry images and quantification results of $CD3^+CD8^+$ T cells in the blood (n=4 mice). Data are presented as the mean ± SD.

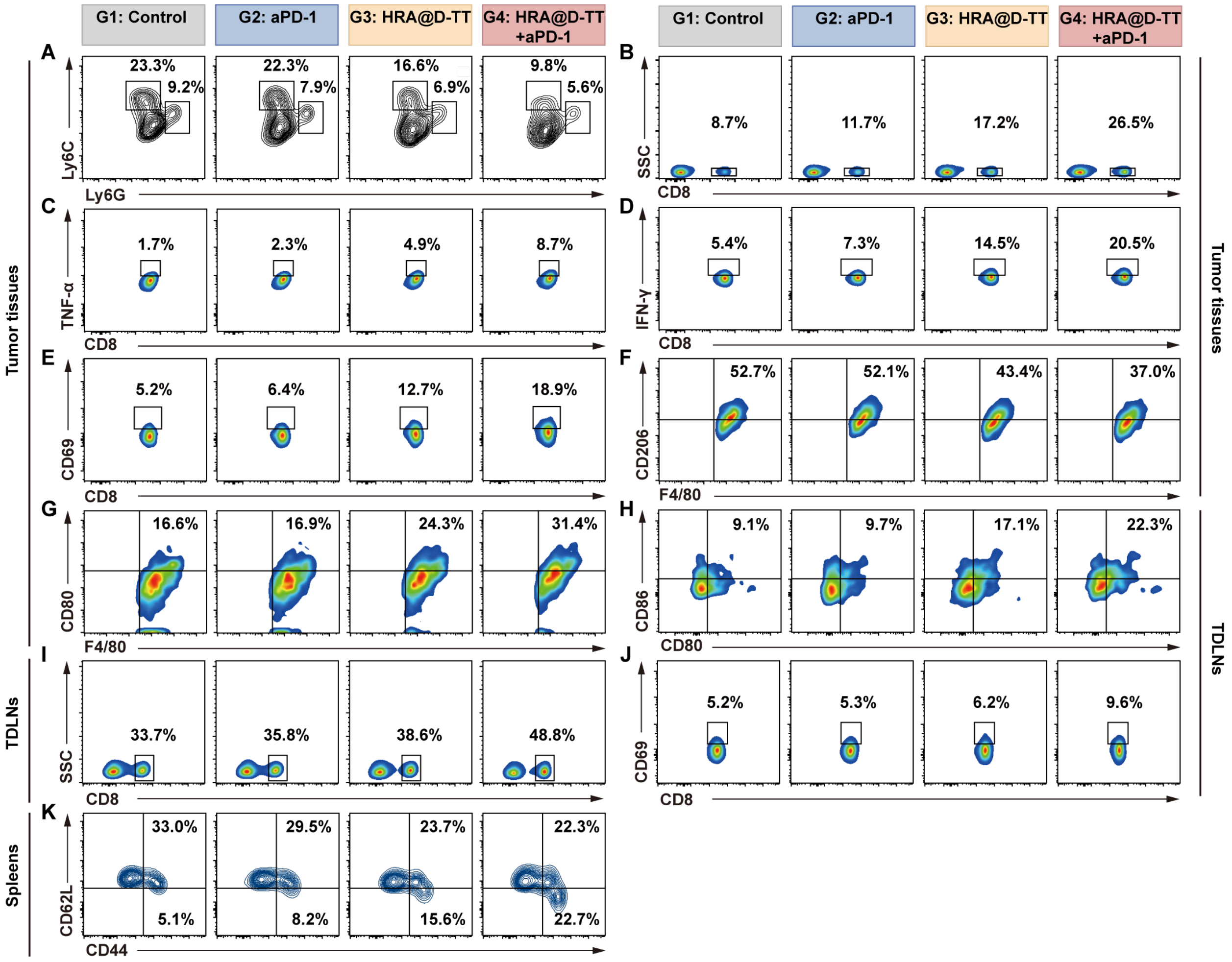

Fig. S33. Representative flow cytometry images of quantitative data in Fig.9. Flow cytometric analysis of **(A)** MDSCs, **(B)** $CD8^+$ T cells, **(C)** TNF-$\alpha^+$$CD8^+$ CTLs, **(D)** IFN-$\gamma^+$$CD8^+$ CTLs, **(E)** $CD69^+CD8^+$ CTLs, **(F)** M2 macrophages, and **(G)** M1 macrophages in tumors. Flow cytometric analysis of the frequency of **(H)** DCs, **(I)** $CD8^+$ T cells, and **(J)** $CD69^+CD8^+$ CTLs in TDLNs. **(K)** Flow cytometric analysis of the T_{EM} in spleens.

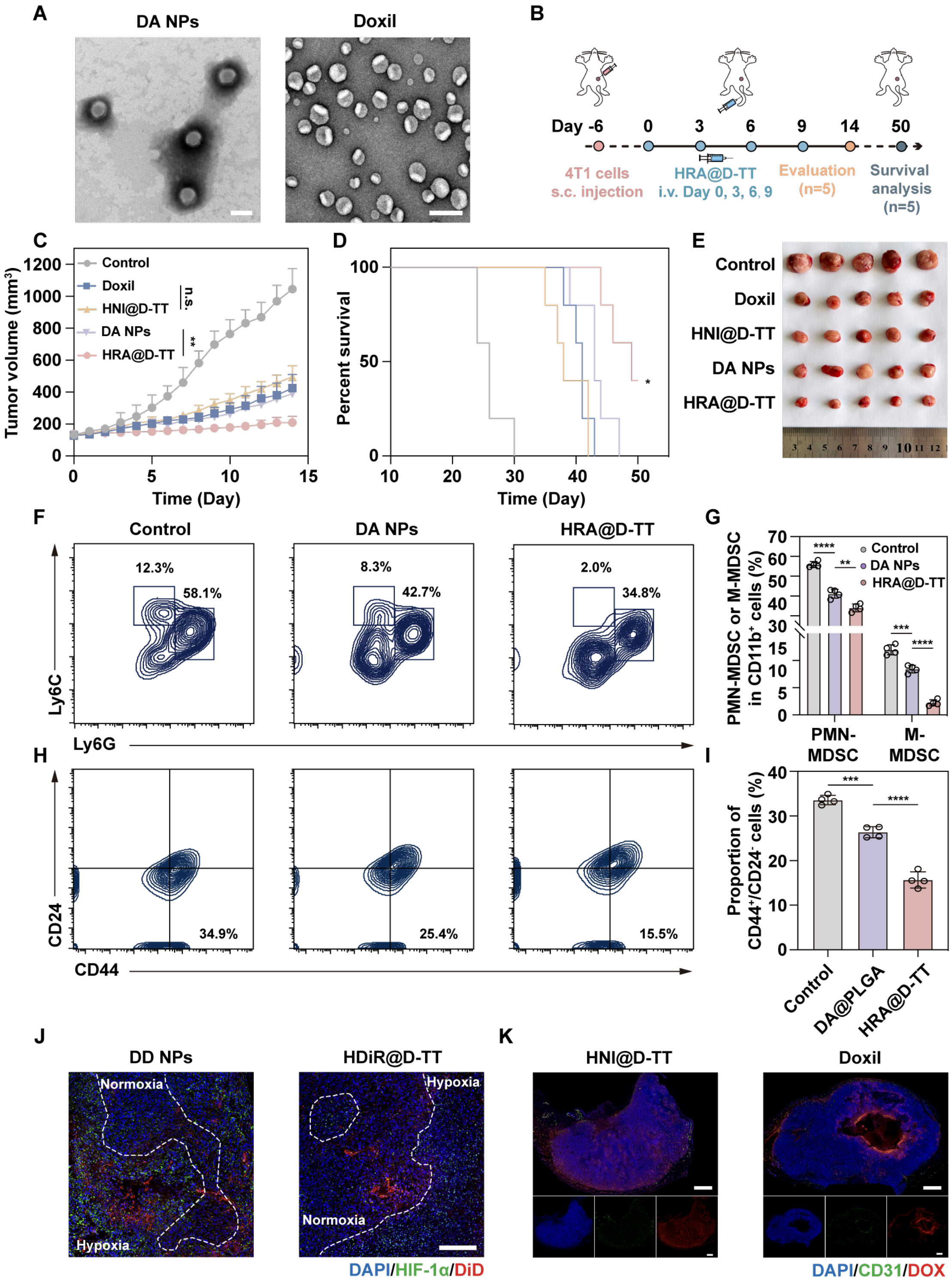

Fig. S34. Comparative analysis of antitumor efficacy among nanoplatforms with distinct drug-delivery mechanisms. (A) TEM images of DA NPs and Doxil. Scale bar represents 200 nm. **(B)** Schema depicting the 4T1 tumor-bearing model construction and treatment schedule. **(C)** Tumor volume, **(D)** survival curves, **(E)** Tumor images of the mice with different treatments (n=5 mice). **(F)** Flow cytometry measurement and **(G)** quantification of $CD11b^{+}Ly6G^{-}Ly6C^{hi}$ M-

MDSCs and $CD11b^+Ly6G^+Ly6C^{low}$ PMN-MDSCs in tumor tissues (n=4 mice). **(H)** Flow cytometry measurement and **(I)** quantification of $CD44^+/CD24^-$ CSCs in tumor tissues (n=4 mice). **(J)** Immunofluorescence staining images of tumor sections from different groups. Scale bar represents 200 μm. **(K)** Representative CLSM images of tumor sections from mice treated with HNI@D-TT and Doxil. Scale bar represents 1 mm. Survival data was analyzed with the log-rank (Mantel-Cox) test. DA NPs versus HRA@D-TT. Other data are represented as mean ± SD and statistical significance was analyzed through one-way analysis of variance (ANOVA). *p < 0.05, **p < 0.01, ***p < 0.001, ****p < 0.0001; n.s. indicates no significance.

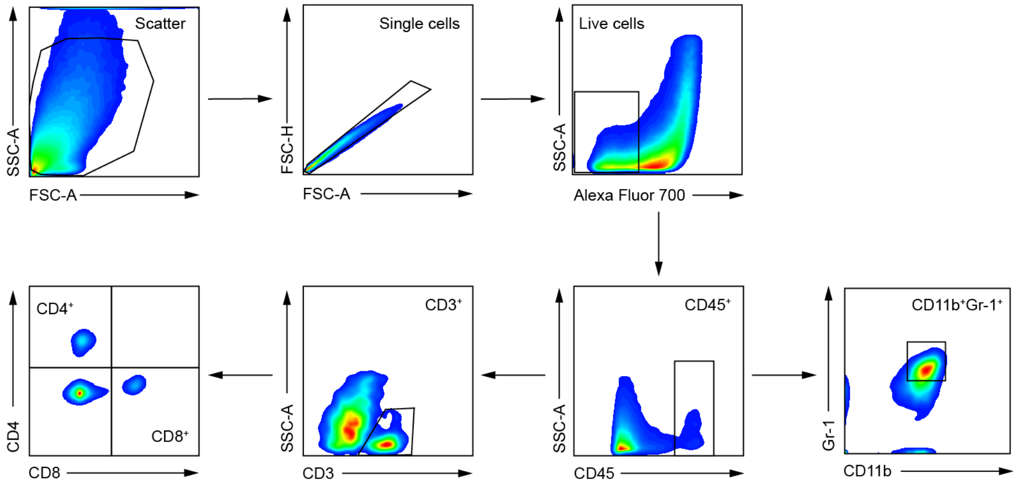

Fig. S35. Gating strategies for CD8$^+$ T cells and CD11b$^+$Gr-1$^+$ MDSCs in tumors.

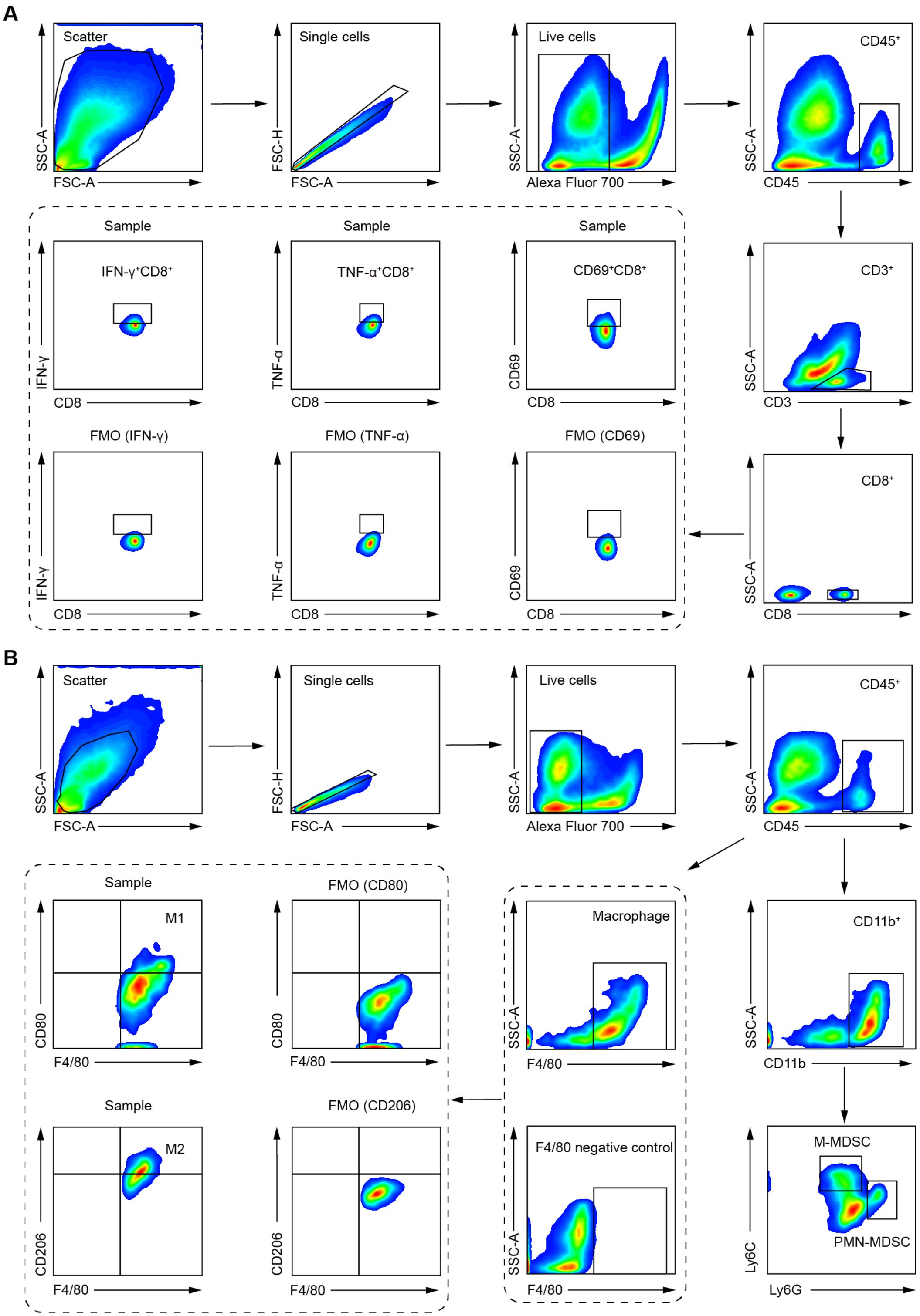

Fig. S36. Gating strategies for tumor-associated immune cell phenotyping. (A) Gating strategies for identifying CD8$^+$ T cells, CD8$^+$CD69$^+$ T cells, CD8$^+$IFN-γ^+ T cells, and CD8$^+$TNF-α^+ T cells in tumor tissues. **(B)** Gating strategies for identifying M1 macrophages, M2 macrophages, M-MDSCs, and PMN-MDSCs in tumor tissues.

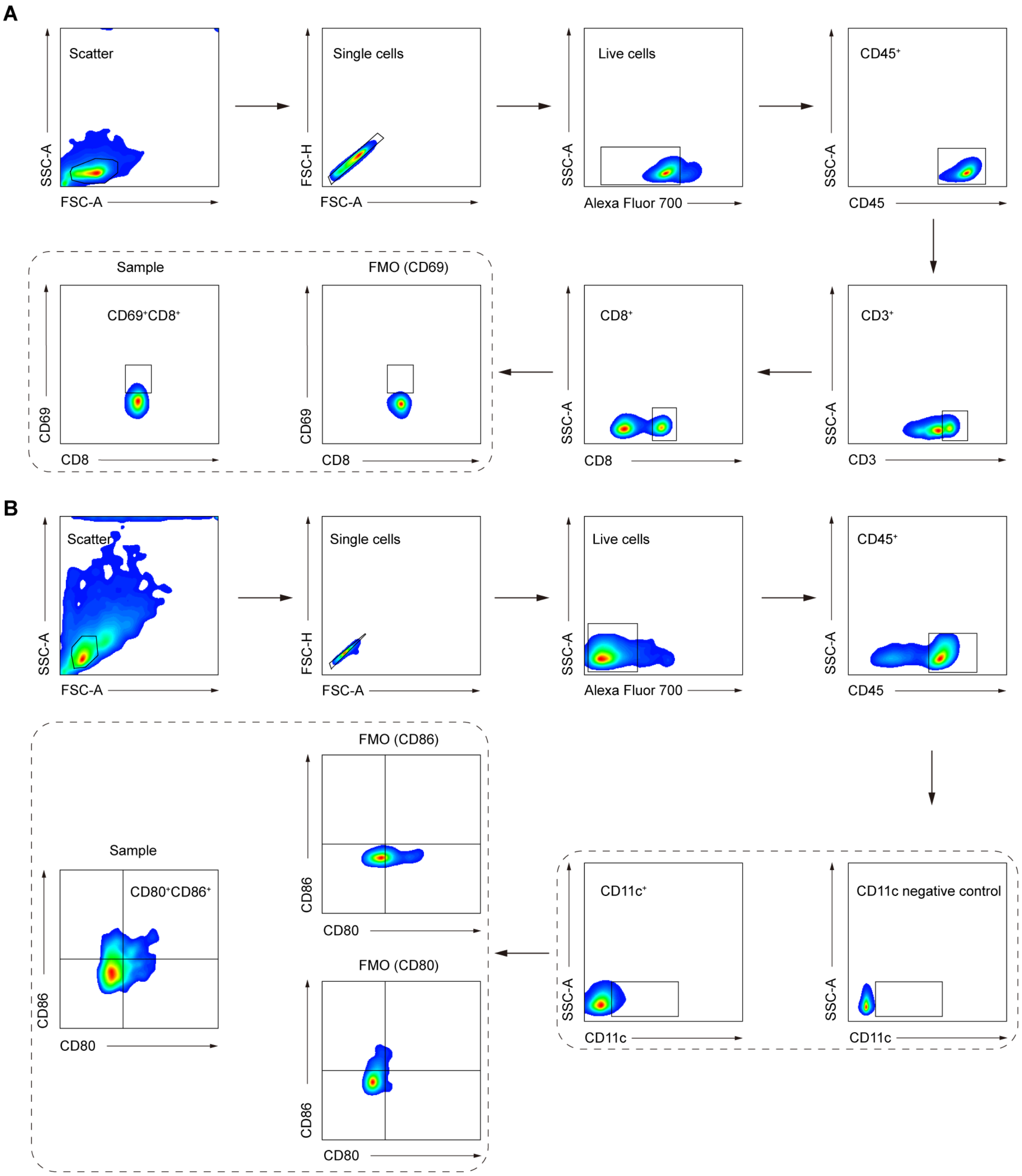

Fig. S37. Gating strategies for identifying immune cells in TDLNs. (A) Gating strategies for $CD8^+$ T cells and $CD8^+CD69^+$ T cells panel in TDLNs. **(B)** Gating strategy for DCs panel in TDLNs.

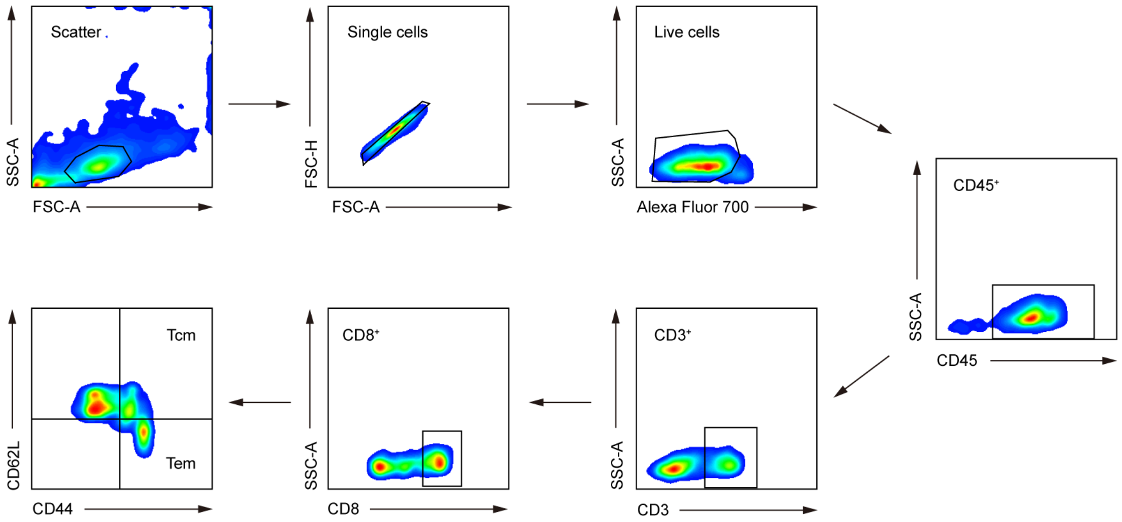

Fig. S38. Gating strategy for identifying T_{EM} in spleens in Fig. S33K.

Table S1. Characterization of HRA and HRA@D-TT.

Nanoparticles	Size (nm)	PDI	Zeta (mV)
HRA	122	0.16	-25.8
HRA@D-TT	161	0.23	-10.5
